# Piezo1 Channel Mediates Mechanically Programmable Drug Delivery to Potentiate Intravesical Chemotherapy

**DOI:** 10.1002/advs.202522936

**Published:** 2026-03-04

**Authors:** Minghai Ma, Xing Li, Minxuan Jing, Zezhong Yang, Jiale He, Jianpeng Li, Xiao Liang, Yunzhong Jiang, Rou Huang, Yuanquan Zhang, Yuanchun Pu, Jiawei Xiu, Yutong Chen, Kaibo Mi, Pu Zhang, Lei Wang, Jinhai Fan

**Affiliations:** ^1^ Department of Urology The First Affiliated Hospital, Xi'an Jiaotong University Xi'an China; ^2^ Department of Thoracic Surgery Tangdu Hospital Air Force Medical University Xi'an China; ^3^ Department of Urology Zhejiang Provincial People's Hospital Hangzhou Medical College Hangzhou China

**Keywords:** bladder cancer, chemotherapy, intravesical instillation, mechanical force, piezo1 channel

## Abstract

Intravesical chemotherapy for bladder cancer remains limited by poor efficacy and significant toxicity, imposing profound physical and psychological burdens on patients. While several physical‐assisted approaches comprising hyperthermic intravesical chemotherapy and electromotive drug administration have been investigated to enhance the chemotherapy, but limited in clinical application due to their technical complexity and high costs. Herein, we introduce a simple yet powerful approach: utilizing programmable mechanical pressure as a therapeutic enhancer to establish a mechano‐chemotherapy strategy. We demonstrate that controlled pressure activates the mechanosensitive ion channel Piezo1 in bladder cancer, triggering a calcium ion cascade that transiently and reversibly amplifies mechanosensitivity and membrane permeability. This force‐controlled process obviously enhances intracellular accumulation of standard chemotherapeutics, including Doxorubicin (DOX) and Mitomycin‐C (MMC), leading to significantly increased tumor cell apoptosis. Crucially, mechano‐chemotherapy potently enhances antitumor efficacy while mitigating dose‐limiting mucosal toxicity in orthotopic models. In a word, this work establishes Piezo1‐mediated mechano‐chemotherapy as a readily translatable strategy, transforming mechanical force into a safe and effective tool for optimizing cancer treatment.

## Graphical abstract

This study utilizes programmable mechanical pressure as a therapeutic enhancer to establish a mechano‐chemotherapy strategy. Controlled pressure activates the mechanosensitive ion channel Piezo1 in bladder cancer, triggering a calcium ion cascade that transiently and reversibly amplifies membrane permeability to chemotherapeutics. Mechano‐chemotherapy potently enhances antitumor efficacy while mitigating dose‐limiting mucosal toxicity, facilitating Piezo1‐mediated mechano‐chemotherapy as a readily translatable strategy.

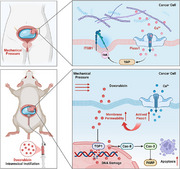

## 1 Introduction

Bladder cancer (BC) ranks as the tenth most commonly diagnosed cancer worldwide, with approximately 614,000 new cases and 220,000 deaths occurring in 2022 [1, 2]. The global age‐standardised incidence rate is 20.0 per 100,000 person‐years for men and 4.6 for women in the European Union [3]. Transurethral resection of bladder tumor (TURBT) is the primary treatment for non‐muscle‐invasive bladder cancer (NMIBC), with postoperative intravesical chemotherapy continually serving as a cornerstone for preventing tumor recurrence [4, 5]. However, the efficacy has been severely hampered by poor drug permeability across the urothelium and dose‐limiting toxicities. High‐concentration chemotherapeutics like Bacillus Calmette‐Guerin (BCG), Mitomycin‐C (MMC), and Doxorubicin (DOX) could induce chemical cystitis, mucosal erosion, edema, bleeding, and systemic complications, imposing profound physical and psychological burdens on patients. Notably, almost 70% of patients experience recurrence or progress within 5 years despite standard therapy, underscoring an urgent need for strategies that enhance drug efficacy while minimizing toxicity as far as possible [6, 7, 8, 9].

In order to improve the efficacy of intravesical chemotherapy, researchers developed several physic‐assisted approaches comprising hyperthermic intravesical chemotherapy, microwave‐induced hyperthermia, conductive chemohyperthermia, and electromotive drug administration (EMDA), etc. [10, 11]. Clinical trials have demonstrated improved recurrence‐free survival (RFS) with hyperthermic MMC or EMDA combined with BCG in high‐risk patients, however these approaches were limited in clinical application due to their technical complexity and high costs [12, 13, 14]. Recently, mechanical forces have become fundamental regulators of organ physiology and pathology [15, 16]. Cellular mechanotransduction, the conversion of mechanical signals into biochemical responses regulating cell activities and metabolism, governs diverse biological processes through stimuli like hydrostatic pressure (HP) [17], fluid shear stress [18], tensile forces [19], matrix stiffness, and fluid viscosity [20], which orchestrate physiological functions such as tissue repair, fibrosis, tumorigenesis, and immunotherapy resistance [21]. The bladder operates within a complex biomechanical stress environment [22]. Mechanical tension, which is generated during bladder filling and emptying, intensifies under bladder outlet obstruction (BOO) due to compensatory hypertrophy and proliferation of bladder cells [23, 24]. HP normally ranging from 0 to 15 cmH_2_O at rest and peaking below 40 cmH_2_O during filling, escalates pathologically to 100–200 cmH_2_O in bladder contracture or urinary retention [25, 26, 27]. Matrix stiffness, inherently heterogeneous across bladder layers, increases markedly during fibrosis via collagen deposition, impairing bladder contractile function [28, 29, 30]. Moreover, the bladder exposed to chemical stressors and biomechanical forces from urine, relies on biochemical and mechanical adaptability to maintain function, enabling cells to preserve structural integrity and metabolic resilience [31]. However, the impact and mechanism of mechanical force on urothelium and drug uptake efficiency in intravesical chemotherapy have never been explored, representing a pivotal knowledge gap in optimizing localized chemotherapy.

Emerging evidence implicates Piezo1, a mechanosensitive ion channel, as a key mediator of cellular mechanical transduction [32, 33, 34, 35, 36]. Luo et al. have reported that intracellular force induced by myosin activates Piezo1 via PI3K/PIP3‐mediated membrane tension, while Piezo1‐regulated Ca^2^^+^ influx enhances force generation via FAK/RhoA/ROCK. This multifunctional feedback circuit identifies Piezo1‐targeting as a therapeutic strategy for bladder disease [37]. Besides, YAP/TAZ activation under elevated hydrostatic pressure drives clathrin‐dependent endocytosis and triggers TEAD‐mediated transcription to regulate components involved in cell volume dynamics and extracellular matrix remodeling, with their absence leading to reduced membrane tension and impaired cellular adaptation to cyclic hydrostatic pressure [38]. These signal responses establish a feedback loop, enabling cells to robustly adjust to fluctuating fluid pressure. Yet the specific mechanism of bladder sensing mechanical pressure during intravesical therapy has been unclear.

**SCHEME 1:**
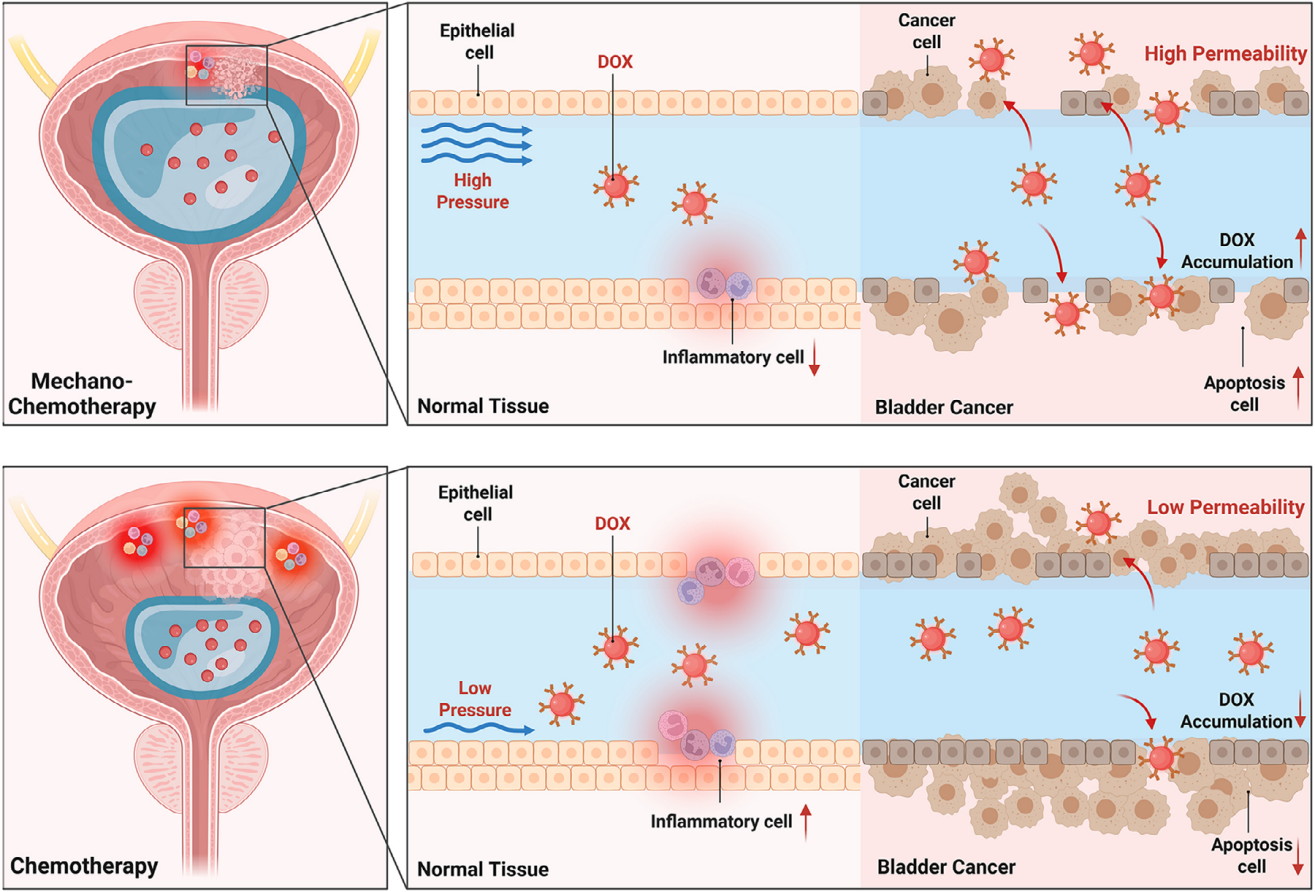
The scheme of mechano‐chemotherapy based on DOX during intravesical instillation in bladder cancer.

Our previous study has implied that upon activation by mechanical force, Piezo1 triggers Ca^2^^+^ influx and collaborates with integrin β1 (ITGB1) to engage extracellular matrix sensing, subsequently driving YAP‐dependent transcriptional reprogramming and endocytosis [39]. In this study, we further investigate whether controlled pressure could exploit mechano‐transduction to force‐prime tumor, augmenting membrane permeability and chemotherapeutic uptake, ultimately forging an effective “mechano‐chemotherapy” synergy to enhance intravesical treatment while mitigating dose‐limiting mucosal toxicity (Scheme 1).

## 2 Results

### 2.1 Mechanical Pressure Potentiates Mechano‐Sensitivity in Urothelium via Piezo1/ITGB1 Activation

**FIGURE 1:**
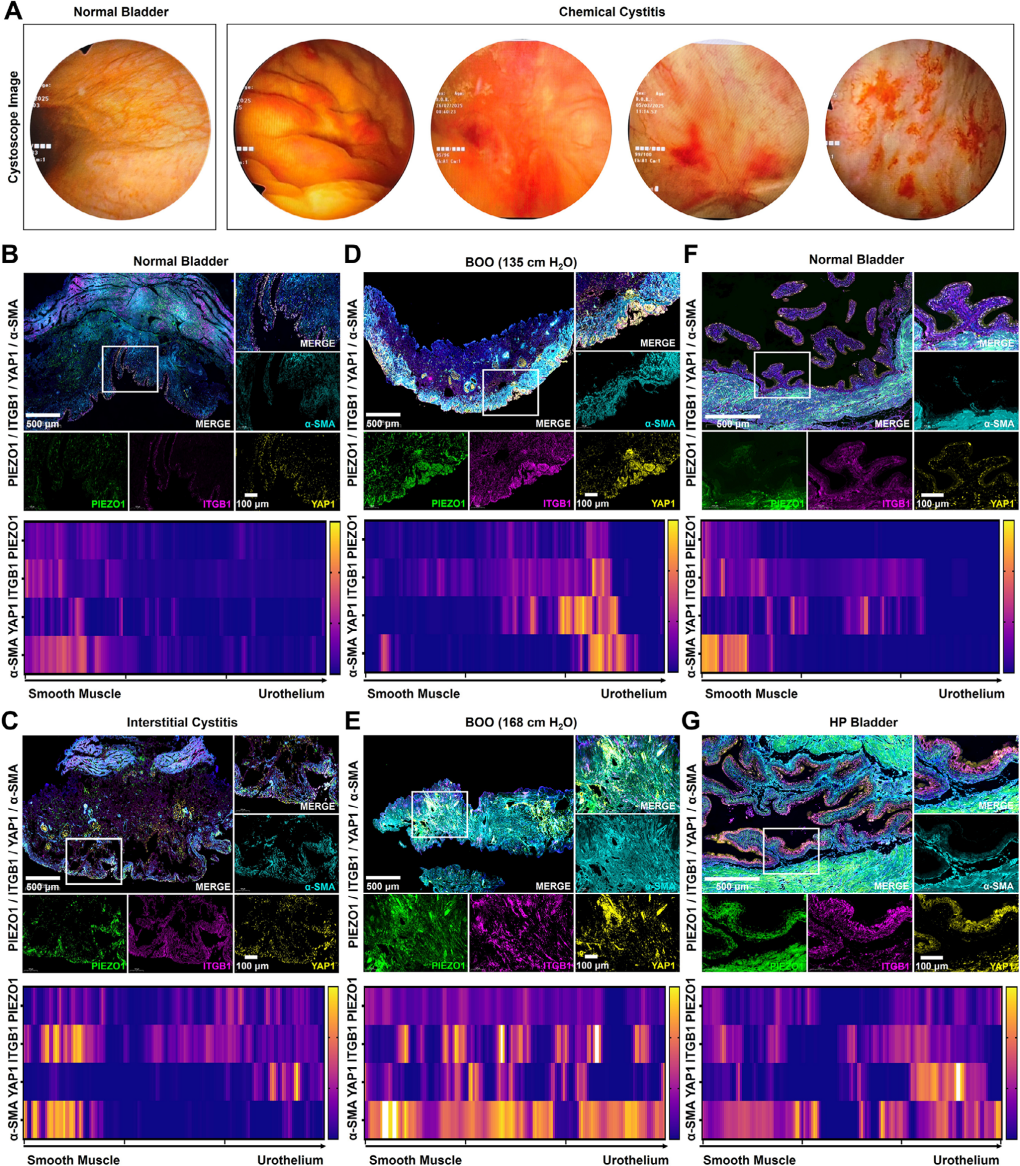
Mechanical pressure enhances mechano‐sensitivity in urothelium via Piezo1/ITGB1 activation. (A) The image from a cystoscope showing chemical cystitis, including mucosal edema, hemorrhage and erosion after long‐term intravesical instillation. (B) mIHC images and quantitative results of mechanical signal Piezo1/ITGB1/YAP1/α‐SMA in human normal bladder. (C) mIHC images and quantitative results of mechanical proteins in the human bladder of interstitial cystitis. (D) mIHC images and quantitative results of mechanical proteins in the human BOO bladder (135 cmH_2_O). (E) mIHC images and quantitative results of mechanical proteins in human BOO bladder (168 cmH_2_O). (F) mIHC images and quantitative results of mechanical proteins in mice normal bladder. G) mIHC images and quantitative results of mechanical proteins in mice bladder after intravesical HP every day for 1 week.

To evaluate the impact of long‐term intravesical therapy on bladder mucosal toxicity, we collected cystoscope images from patients treated with BCG and chemotherapeutic agents. The results illustrated that long‐term intravesical instillation post‐bladder tumor resection induced severe chemical cystitis, including mucosal edema, hemorrhage and erosion, causing significant patient suffering and diminishing quality of life (Figure 1A). To further investigate the impact of mechanical pressure on the bladder urothelium, multiplex immunohistochemical (mIHC) analysis of human and mice bladder tissues was involved. The phenomenon revealed significant upregulation and prominent co‐localization of mechanical signal axis Piezo1/ITGB1/YAP1, and alpha smooth muscle actin (α‐SMA) in the bladder tissues of interstitial cystitis patients compared to normal people, particularly within the urothelium regions (Figure 1B,C). Furthermore, in the BOO bladder (BOO index: 135 cmH_2_O and 168 cmH_2_O), Piezo1/ITGB1 exhibited aberrant spatial redistribution from initial muscularis localization toward the mucosal epithelium, indicating that mechanical pressure promotes spatial reorganization of the mechanical signal to enhance urothelial mechanosensitivity possibly (Figure 1D,E). We subsequently confirmed that mechanical pressure also significantly increased Piezo1/ITGB1 expression with enhanced spatial localization within the urothelial layer of mice bladder after intravesical mechanical pressure every day for 1 weeks (Figure 1F,G). These findings collectively demonstrated that Piezo1/ITGB1 axis served as a key regulator for urothelial to modulate mechanical microenvironment, driving spatial reconfiguration of mechanosensitive proteins. However, the precise regulatory mechanisms remain elusive and need to be further investigated.

### 2.2 Mechanical Pressure Enhances Mechano‐Sensitivity of Urinary Epithelial Cells

**FIGURE 2:**
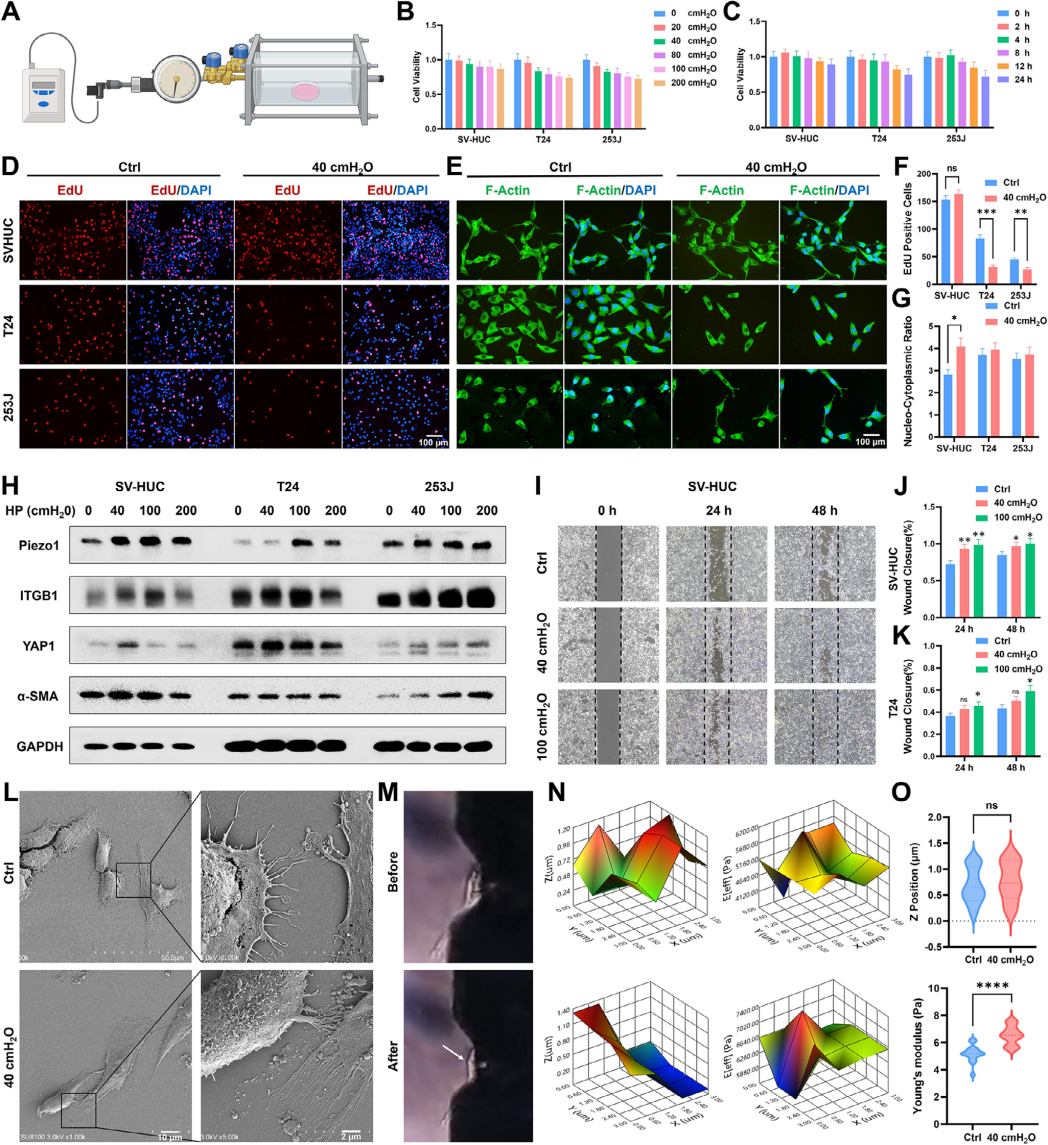
Mechanical pressure enhances mechano‐sensitivity of urinary epithelial cells. (A) The scheme of mechanical pressure model in vitro. B) CCK‐8 assay of SV‐HUC, T24, 253J cells exposed on gradient pressure. (C) CCK‐8 assay of cells exposed on 40 cmH_2_O pressure for different times. (D) EdU assay of cells exposed on 40 cmH_2_O pressure for 12 h. (E) F‐Actin staining of SV‐HUC, T24, 253J cells exposed on 40 cm H_2_O pressure for 12 h. (F) The quantitative analysis of EdU assay. (G) The quantitative analysis of nucleo‐cytoplasmic ratio. (H) Expression level of Piezo1/ITGB1/YAP/α‐SMA in cells exposed on gradient pressure. (I) Wound closure assay of SV‐HUC cells exposed on gradient pressure for 24, 48 h. (J) The quantitative analysis of wound closure assay in SV‐HUC cells. (K) The quantitative analysis of wound closure assay in T24 cells. (L) TEM image of SV‐HUC cell exposed on 40 cm H_2_O pressure. (M) The detection process of nanoindentation before and after HP for 2 h. N) Detection of Young's modulus in T24 cells using nanoindentation before and after HP for 2 h. (O) The quantitative analysis of Z position and Young's modulus detection. All data are presented as the Mean ± SD (*n* = 3). ^*^ *p <* 0.05, ^**^ *p <* 0.01, and ^***^ *p <* 0.001. ns, no significant difference.

To further investigate the impact of mechanical pressure on bladder urothelial and tumor cells, we established an in vitro hydrostatic pressure model enabling precise pressure regulation from 0 to 200 cmH_2_O (Figure 2A). Gradient pressure elevation significantly inhibited the growth of bladder cancer cells T24 and 253J while minimally affecting urothelial cells SV‐HUC (Figure 2B). Pressure at 40 cmH_2_O markedly inhibited tumor cell growth after 12 h exposure (Figure 2C). This finding corroborated by EdU assays demonstrating 40 cmH_2_O pressure inhibited tumor proliferation without affecting normal urothelial cells (Figure 2D). Concurrently, mechanical pressure remodeled F‐actin cytoskeletal structure and increased nuclear‐to‐cytoplasmic ratios especially in SV‐HUC cells, resulting in elongated cell morphology (Figure 2E). The quantitative results were shown in Figure 2F,G. Moreover, western blotting results exhibited that key mechanosensitive proteins (Piezo1, ITGB1, YAP1, α‐SMA) exhibited pressure‐dependent upregulation even at 40 cmH_2_O pressure (Figure 2H). Notably, mechanical pressure enhanced urothelial cell migration but minimally altered T24 and 253J tumor cell motility (Figure 2I–K; Figure S1). Transmission electron microscopy (TEM) further revealed pressure‐promoted microfilament assembly and heightened adhesion in SV‐HUC cells (Figure 2L). Moreover, nanoindentation further demonstrated increased cellular stiffness in T24 cells (Figure 2M,N), with Young's modulus elevating from 5.2 kPa to 6.5 kPa under pressure (Figure 2O). These results collectively indicate that mechanical pressure promotes urothelial adhesion and migration while augmenting cellular rigidity, thereby potentiating mechanosensitivity.

### 2.3 Mechanical Pressure Mediates Transcriptional Reprogramming to Amplify Calcium Influx

**FIGURE 3:**
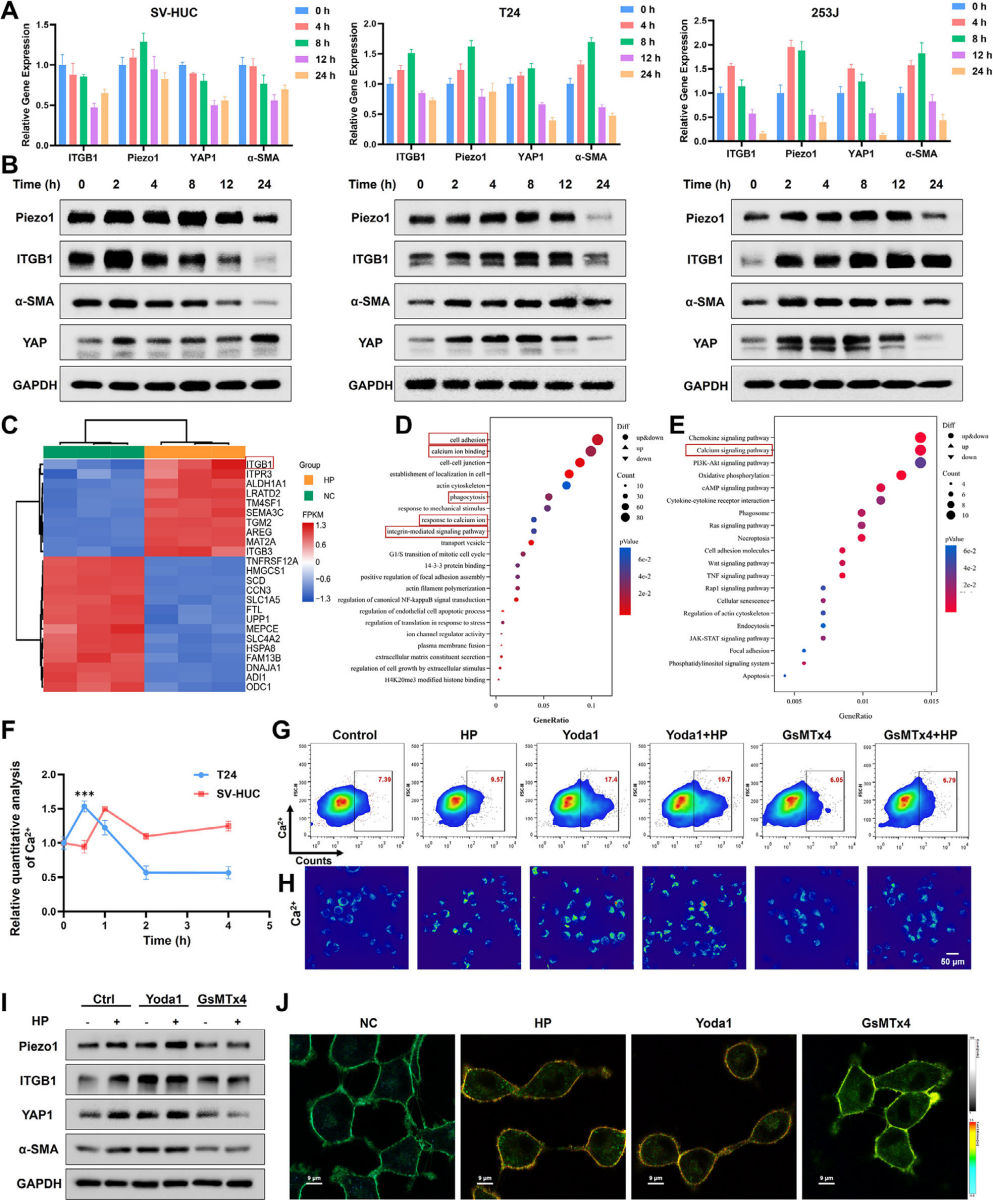
Mechanical pressure amplifies calcium influx via Piezo1/ITGB1‐mediated membrane tension. (A) RNA expression level of Piezo1/ITGB1 in cells exposed on mechanical pressure. (B) Protein expression level of Piezo1/ITGB1 in cells exposed on hydrostatic pressure. (C) The heatmap of differentially expressed genes (DEGs) in T24 cells from RNA sequencing. (D) Go analysis of DEGs in T24 cells from RNA sequencing. E) KEGG analysis of DEGs in T24 cells from RNA sequencing. (F) The flow cytometry result of intracellular calcium flux in T24 and SV‐HUC cells exposed on hydrostatic pressure for 0, 0.5, 1, 2, and 4 h using Fluo‐4AM probe. (G) The flow cytometry result of intracellular calcium flux in T24 cells exposed on hydrostatic pressure with Piezo1 agonists or inhibitors. (H) The image of intracellular calcium flux in T24 cells exposed on hydrostatic pressure with agonists or inhibitors using Fluo‐4AM probe. (I) Expression level of Piezo1/ITGB1 signal of cells exposed on pressure with agonists or inhibitors. (J) Membrane tension through fluorescence lifetime imaging (FLIM) in T24 cells exposed on pressure for 2 h. All data are presented as the Mean ± SD (*n* = 3). ^*^ *p <* 0.05, ^**^ *p <* 0.01, and ^***^ *p <* 0.001. ns, no significant difference.

To delineate pressure‐induced mechanotransduction at the transcriptional level, we quantified RNA and protein expression dynamics via RT‐qPCR and Western blotting in SV‐HUC, T24, 253J cells. The results exhibited that hydrostatic pressure (40 cm H_2_O) upregulated Piezo1, ITGB1, YAP1, and α‐SMA within 8 h in tumor cells T24 and 253J (Figure 3A), with corresponding protein elevations peaking at 12 h followed by partial downregulation, demonstrating revertible mechanosensitive gene activation (Figure 3B). Notably, urothelial cells exhibited little upregulation of mechanical signal, suggesting heightened mechanosensitivity and responsiveness to mechanical stimuli in tumor cells. In order to clarify the specific mechanism, RNA‐sequencing revealed pronounced upregulation of differentially expressed genes (DEGs), including adhesion molecules ITGB1 and so on (Figure 3C) in T24 cell, corroborated by GO enrichment analyses highlighting enhanced cellular adhesion, calcium signaling, and phagocytosis (Figure 3D). KEGG pathway analysis further identified upregulated cytokine and calcium signaling pathways (Figure 3E).

Then we detected the intracellular calcium flux using Fluo‐4AM probe, and the results confirmed tumor cells exhibited a more rapid and pronounced Ca^2^^+^ response, peaking at 0.5 h post‐pressure, whereas normal cells showed a delayed and attenuated peak (Figure 3F). The flow cytometry results were exhibited in Figure S2, suggesting a potential temporal window for selective tumor targeting. Pharmacological modulation using Yoda1 (Piezo1 agonist) and GsMTx4 (Piezo1 inhibitor) established that Yoda1‐enhanced and GsMTx4‐suppressed Ca^2+^ mobilization under mechanical pressure from flow cytometry (Figure 3G), also validated by confocal Ca^2+^ imaging (Figure 3H). Yoda1 potentiated pressure‐induced Piezo1/ITGB1 axis expression, whereas GsMTx4 abolished this effect (Figure 3I). Crucially, fluorescence lifetime imaging (FLIM) revealed pressure‐elevated membrane tension, which Yoda1 augmented and GsMTx4 attenuated (Figure 3J).

Further validating the impact of mechanical pressure on protein expression and function, our proteomic sequencing revealed multiple differentially expressed proteins (DEPs) in pressure‐treated cells (Figure S3A,B), with a predominant nuclear localization indicating enhanced nuclear responsiveness to mechanical signals (Figure S3C). Subsequent functional annotation demonstrated that these DEPs were significantly enriched in biological processes related to the myosin complex and calcium ion signaling (Figure S3D). KEGG pathway analysis further identified mechano‐sensitive enrichment in focal adhesion, endocytosis, cytoskeletal reorganization, and calcium signaling pathways (Figure S3E). Collectively, these multi‐omics findings strengthen our mechanistic framework by demonstrating that mechanical loading transcriptionally reprograms genes coordinating cytoskeletal dynamics and calcium flux, thereby corroborating Piezo1/ITGB1 mechanotransduction axis and triggering membrane tension amplification to potentiate adhesion and phagocytic functions.

### 2.4 Mechanical Pressure Enhances the Accumulation of Chemotherapeutic Agent DOX in Bladder Tumor

**FIGURE 4:**
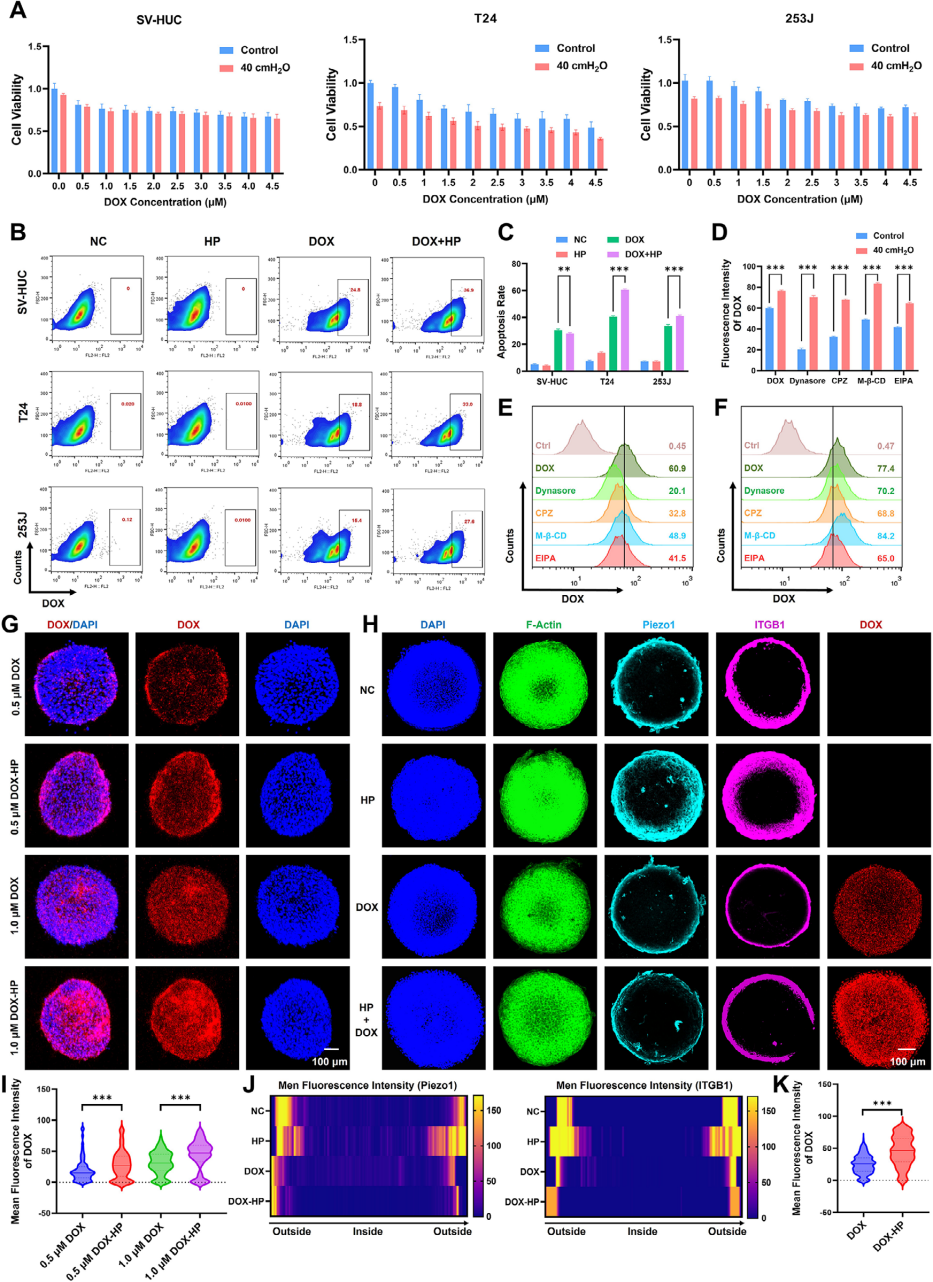
Mechanical pressure enhances DOX accumulation in tumor. (A) Proliferation assay of SV‐HUC, T24, 253J cells exposed on 40 cm H_2_O pressure and different concentrations of DOX. (B) The uptake efficiency of DOX in cells exposed on HP from flow cytometry. (C) The quantitative analysis of the apoptosis rate of cells induced by DOX and HP from flow cytometry. D)The quantitative result of DOX uptake efficiency from flow cytometry using a series of endocytic inhibitors. (E) DOX uptake efficiency of T24 cells exposed on DOX from flow cytometry using a series of endocytic inhibitors. (F) DOX uptake efficiency of T24 cells exposed on DOX and HP from flow cytometry using a series of endocytic inhibitors. (G) DOX accumulation in tumor spheres exposed on HP and different concentrations of DOX. H) Immunofluorescence of Piezo1/ITGB1 in tumor spheres exposed on HP and DOX. I) The quantitative analysis of DOX intensity in tumor spheres treated with different concentrations of DOX. J) The quantitative analysis of Piezo1 and ITGB1 expression levels in tumor spheres. K) The quantitative analysis of DOX accumulation in tumor spheres treated with DOX and HP. All data are presented as the Mean ± SD (*n* = 3). ^*^ *p <* 0.05, ^**^ *p <* 0.01, and ^***^ *p <* 0.001. ns, no significant difference.

Building on the evidence that mechanical pressure augments endocytic capacity, we further investigated whether mechanical force potentiates chemotherapeutic agent DOX uptake in bladder cancer. Cell proliferation assays were first utilized to ensure the proper concentration of 1.0 µm of DOX. 40 cm H_2_O and 100 cm H_2_O significantly enhanced DOX‐mediated cytotoxicity, indicating heightened chemosensitivity (Figure 4A; Figure S4). Flow cytometry further confirmed that mechanical pressure drove DOX uptake and accumulation in T24 and 253J cells (Figure 4B; Figure S5). Besides, mechanical pressure could enhance cell apoptosis induced by DOX significantly in T24 and 253J cells (Figure 4C; Figure S6). Mechanistic interrogation using a series of endocytic inhibitors (EIPA: Macropinocytosis; CPZ: Clathrin‐mediated endocytosis, M‐𝛽‐CD: Caveolin‐mediated endocytosis; Dynasore: Dynamin‐mediated endocytosis) revealed that initial DOX internalization relies mostly on dynamin‐dependent and clathrin‐mediated pathways, whereas pressure‐induced uptake operated possibly independent of classical endocytosis, primarily increasing in membrane permeability instead (Figure 4D). The flow cytometry result was exhibited in Figure 4E,F. This phenomenon suggested that mechanical pressure might enhance membrane permeability and passive diffusion to facilitate DOX accumulation, which was also corroborated in 3D tumor spheres, where mechanical pressure promoted DOX penetration into the inside (Figure 4G). Immunofluorescence of tumor spheres further revealed outside‐in activation of Piezo1/ITGB1 from the periphery inward, spatially correlating with DOX accumulation possibly (Figure 4H). The quantitative data were exhibited in Figure 4I–K. Collectively, mechanical pressure promotes the passive transport of DOX through increased membrane permeability possibly, then driving intracellular drug accumulation and apoptosis to amplify chemosensitivity.

To further determine whether mechanical pressure enhance non‐selective membrane permeability through Piezo1 activation, we performed an uptake assay using Calcein‐AM, serving as an indicator of non‐selective membrane permeability. Mechanical pressure significantly increased Calcein‐AM uptake, and the effect was markedly attenuated by Piezo1 inhibitor GsMTx4. Conversely, application of Piezo1 agonist Yoda1 mimicked the effect of mechanical pressure (Figure S7). Besides, the pressure‐induced increase in membrane permeability started to revert toward baseline levels after the cessation of HP for 2 h (Figure S8). Therefore, Piezo1 activation is necessary for mediating pressure‐induced increases in membrane permeability transiently and reversibly, occurring independently of plasma membrane injury.

### 2.5 Mechanical Pressure Enhances Tumor Apoptosis and Chemotherapeutic Efficacy Through Piezo1 Channel

**FIGURE 5:**
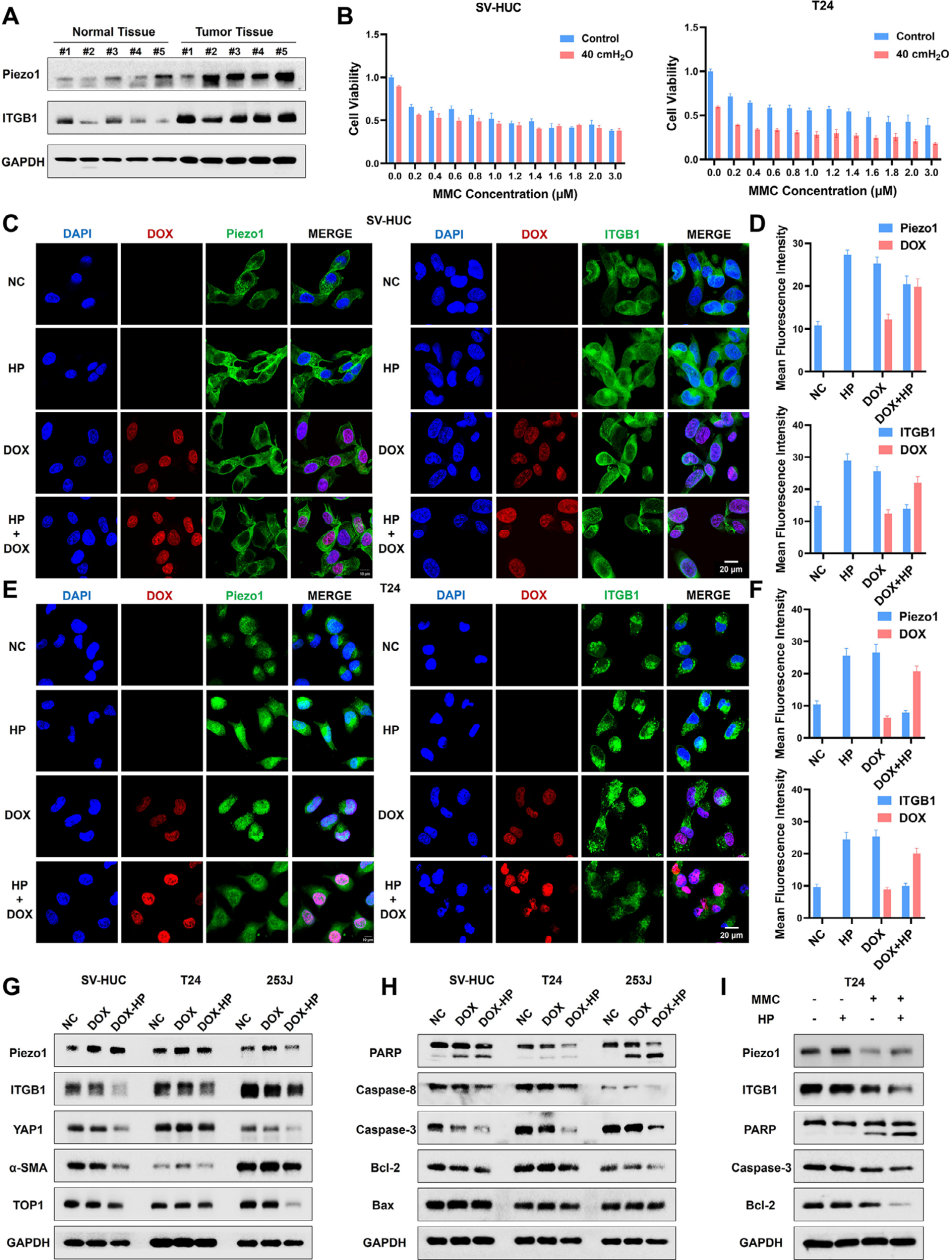
Mechanical pressure enhances tumor apoptosis induced by chemical drugs. (A) Western blotting results of Piezo1/ITGB1 expression level in five matched pairs of bladder normal and tumor tissues. (B) CCK‐8 assay of T24 cell exposed on 40 cm H_2_O pressure and different concentrations of MMC. (C) Immunofluorescence of Piezo1/ITGB1 in SV‐HUC cells exposed on HP and DOX. (D) The quantitative analysis of Piezo1/ITGB1 expression and DOX nuclear accumulation in SV‐HUC cells. (E) Immunofluorescence of Piezo1/ITGB1 in T24 cells exposed on HP and DOX. (F) The quantitative analysis of Piezo1/ITGB1 expression and DOX nuclear accumulation in T24 cells. (G,H) Expression level of mechanical axis and apoptosis proteins in cells treated with DOX and mechanical pressure. (I) Expression level of apoptosis proteins in T24 cells treated with MMC and mechanical pressure.

To elucidate the role of the Piezo1 channel in chemotherapeutic drug uptake, we first analyzed five matched pairs of bladder normal tissue and tumor tissue. The western blotting results revealed a significant upregulation of both Piezo1 and ITGB1 in tumors (Figure 5A), suggesting that tumor cells possess a heightened mechanosensitivity that could facilitate force‐enhanced drug delivery. To further investigate whether the mechano‐chemotherapy strategy was specific to doxorubicin or represented a broader principle, we tested its combination with Mitomycin C (MMC), another frontline intravesical drug for bladder cancer. Consistent with DOX, the application of HP significantly potentiated the cytotoxicity of MMC in tumor cells, but was markedly less pronounced in normal urothelial cells (Figure 5B), demonstrating that mechanical pressure can broadly enhance the efficacy of intravesical chemotherapeutics. Then, immunofluorescence imaging revealed that mechanical pressure upregulated Piezo1/ITGB1 expression in both normal (Figure 5C,D) and tumor cells (Figure 5E,F). While pressure enhanced nuclear DOX accumulation, it concurrently downregulated Piezo1/ITGB1 protein levels, with significantly greater DOX enrichment observed in T24 cells. Western blotting analysis further demonstrated that pressure‐DOX treatment markedly suppressed expression of mechanosensitive proteins and DNA topoisomerase I (TOP1), which inhibited DNA replication (Figure 5G). Besides, both DOX‐HP and MMC‐HP inhibited PARP, caspase‐3, and Bcl‐2 to promote tumor apoptosis (Figure 5H,I). These findings establish that mechanical force acts as a broad delivery enhancer that initiates Piezo1/ITGB1 signaling to drive drug accumulation, disrupting DNA replication, and inducing caspase‐dependent apoptosis.

**FIGURE 6:**
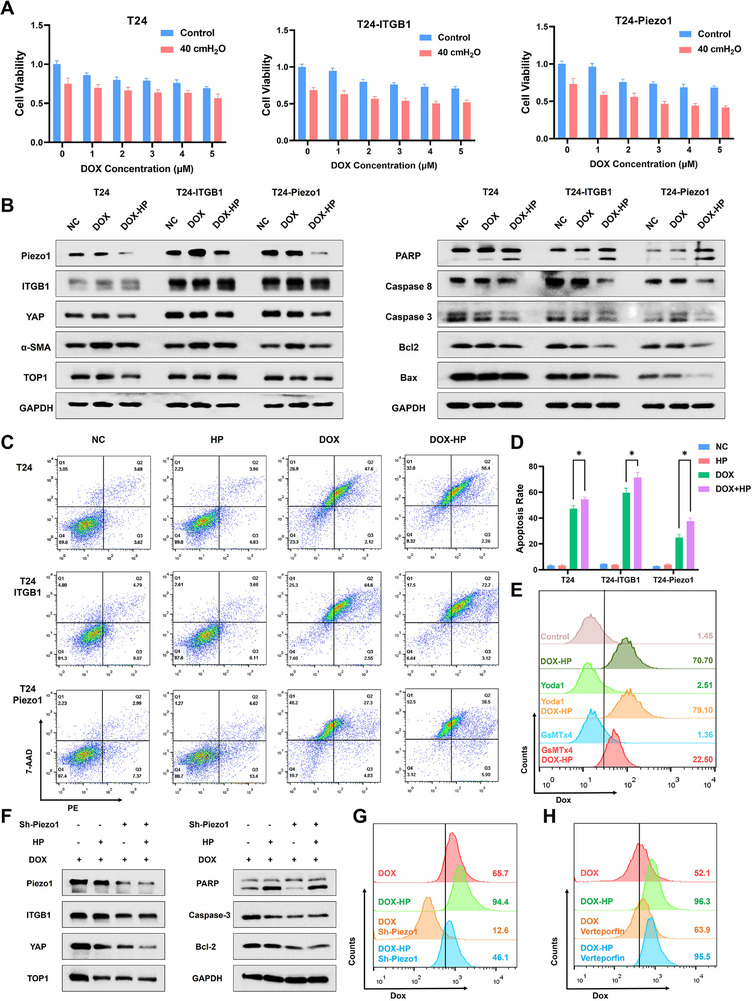
Piezo1 mediates mechanical potentiation of chemotherapeutic efficacy. (A) Proliferation assay of overexpressed cells exposed on HP and different concentrations of DOX. (B) Expression level of the mechanical pathway and apoptosis proteins in cells treated with DOX and HP. (C) The apoptosis rate induced by DOX and HP from flow cytometry. (D) The quantitative analysis of apoptosis rate from flow cytometry. (E) The flow cytometry result of DOX accumulation in T24 cells exposed on HP with Piezo1 agonists or inhibitors. (F) Expression level of the mechanical pathway and apoptosis proteins in Sh‐Piezo1 cells treated with DOX and HP. (G) The flow cytometry result of DOX accumulation in Sh‐Piezo1 cells exposed on HP. (H) The flow cytometry result of DOX uptake in T24 cells treated with YAP‐specific inhibitor verteporfin. All data are presented as the Mean ± SD (*n* = 3). ^*^ *p <* 0.05, ^**^ *p <* 0.01, and ^***^ *p <* 0.001. ns, no significant difference.

To further establish the causal relationship between Piezo1 signaling and apoptosis induced by DOX, we generated stable Piezo1‐overexpressed and Piezo1‐knockdown cell lines (Figure S9A) via lentiviral transduction. Proliferation assays confirmed that Piezo1/ITGB1 overexpression heightened cellular sensitivity to mechanical pressure, significantly potentiating pressure‐enhanced DOX cytotoxicity (Figure 6A). Western blotting further revealed that DOX‐HP treatment in overexpressed cells robustly activated PARP cleavage while suppressing anti‐apoptotic proteins (caspase‐8/3, Bcl‐2), though Piezo1/ITGB1 overexpression partially mitigated DOX‐induced apoptosis (Figure 6B). Then, flow cytometry results demonstrated that Piezo1/ITGB1‐overexpressed cells exhibited increased apoptosis under DOX‐HP treatment, unequivocally validating Piezo1/ITGB1's essential role in mechano‐dependent drug uptake (Figure 6C,D). Complementary pharmacological modulation using the Piezo1 agonist Yoda1 and inhibitor GsMTx4 further corroborated this mechanism, where GsMTx4 reduced pressure‐driven DOX accumulation significantly (Figure 6E). The quantitative data were exhibited in Figure S9B. However, in Piezo1‐knockdown cells (Sh‐Piezo1), the combined treatment of DOX and HP failed to significantly alter the expression levels of TOP1 or key apoptosis‐related proteins (Caspase‐3, Bcl‐2), contrasting with the marked changes observed in Piezo1‐overexpressed cells (Figure 6F). Concomitantly, the results from flow cytometry illustrated that Piezo1 knockdown markedly attenuated the pressure‐induced increase in intracellular DOX accumulation (Figure 6G). Furthermore, treatment with the YAP‐specific inhibitor verteporfin did not significantly inhibit DOX uptake under mechanical pressure (Figure 6H). The quantitative results from flow cytometry were exhibited in Figure S9C,D. And the fluorescence images of DOX accumulation in sh‐Piezo1 cells were shown in Figure S10. Collectively, these results collectively demonstrate that Piezo1, rather than its downstream effector YAP, acts as the master regulatory axis for mechanical potentiation of chemotherapeutic efficacy, orchestrating enhanced drug uptake, nuclear accumulation, and apoptotic execution, thereby establishing a mechano‐chemotherapy synergy to improve cancer treatment.

### 2.6 Mechano‐Chemotherapy Enables Dose Reduction While Sustaining Efficacy and Attenuating Toxicity

**FIGURE 7:**
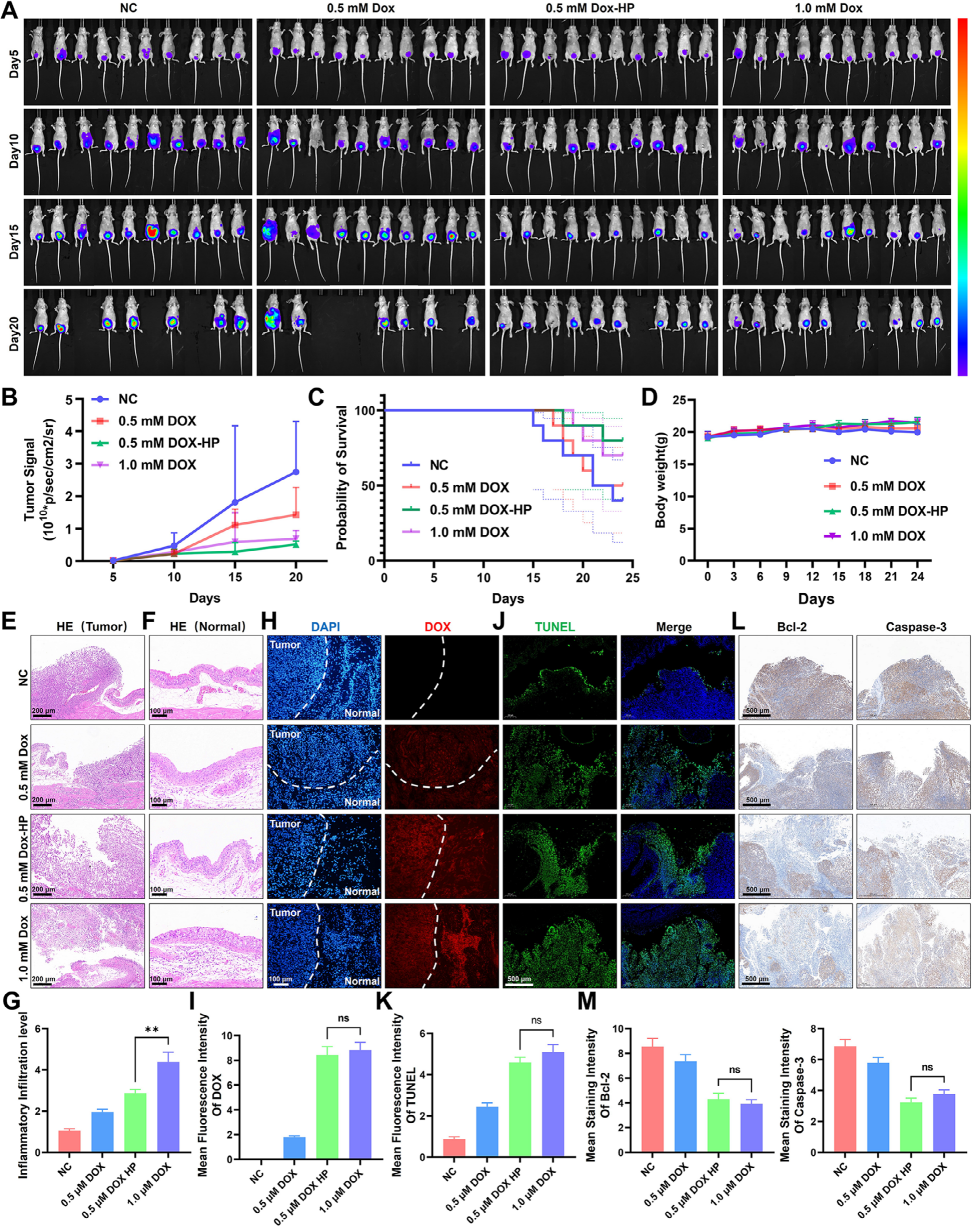
Mechano‐chemotherapy enables dose reduction while sustaining efficacy and attenuating toxicity. (A) IVIS image of orthotopic bladder tumor‐bearing mice received DOX intravesical instillation after mechanical activation. (B) The tumor signal of various groups from IVIS. (C) The survivorship curve of mice in different groups. (D) The body weight of mice in different groups. (E) H&E image of tumor tissues in different groups. F) H&E image of bladder mucosal epithelium in different groups. (G) The quantitative data of the inflammatory infiltration level in submucous layer. (H) Fluorescence image of DOX accumulation in tumor areas. (I) The quantitative data of DOX fluorescence intensity in tumor areas. (J) TUNEL staining image of tumor tissues in different groups. (K) The quantitative data of TUNEL intensity in tumor areas. (L) IHC staining of Bcl‐2 and Caspase‐3 expression in tumor areas. (M) The quantitative data of Bcl‐2 and Caspase‐3 expression level. All data are presented as the Mean ± SD (*n* = 3). ^*^ *p <* 0.05, ^**^ *p <* 0.01, and ^***^ *p <* 0.001. ns, no significant difference.

To establish a physiologically relevant pressure parameter for subsequent intravesical treatment, we first characterized the murine bladder pressure‐volume relationship. The results demonstrated a linear increase in intravesical pressure up to an infusion volume of 200 µL (90 cm H_2_O), indicating maximal bladder wall tension under the condition. Then the infusion volume of 100 µL, which generated 40 cm H_2_O pressure, was selected for subsequent chemotherapeutic instillation experiments (Figure S11). To validate the therapeutic efficacy of mechanical pressure in vivo, orthotopic bladder tumor‐bearing mice received intravesical DOX after HP treatment, with tumor progression monitored by in vivo IVIS imaging (Figure 7A). Critically, 0.5 mM DOX after mechanical pressure achieved comparable antitumor efficacy to 1.0 mM DOX alone, evidenced by equivalent reductions in tumor fluorescence intensity (Figure 7B) and comparable survival rates (Figure 7C). DOX‐pressure treatment caused no significant weight loss (Figure 7D). Histopathological analysis further revealed extensive necrosis and apoptosis with tumor disintegration in both 0.5 mm DOX‐HP and 1.0 mm DOX groups (Figure 7E). However, the high‐dose DOX group (1.0 mm) induced severe cystitis, characterized by mucosal vacuolization, epithelial atypia, and eosinophil‐dense lamina propria infiltration, whereas the 0.5 mm DOX‐HP group exhibited markedly attenuated inflammation (Figure 7F). The quantitative result of the inflammatory infiltration level in the submucous layer was exhibited in Figure 7G. Consistently, pressure‐enhanced DOX delivery elevated tumor accumulation in the 0.5 mM DOX‐HP group to levels indistinguishable from 1.0 mm DOX (Figure 7H,I). TUNEL staining further confirmed pressure‐DOX synergy significantly augmented apoptosis (Figure 7J,K), while immunohistochemistry demonstrated concomitant suppression of Bcl‐2 and caspase‐3 activation in treated tumors (Figure 7L,M). These findings establish that mechano‐chemotherapy synergistically enhances DOX accumulation and apoptotic execution in tumor area while mitigating dose‐limiting mucosal toxicity.

## 3 Discussion

**FIGURE 8:**
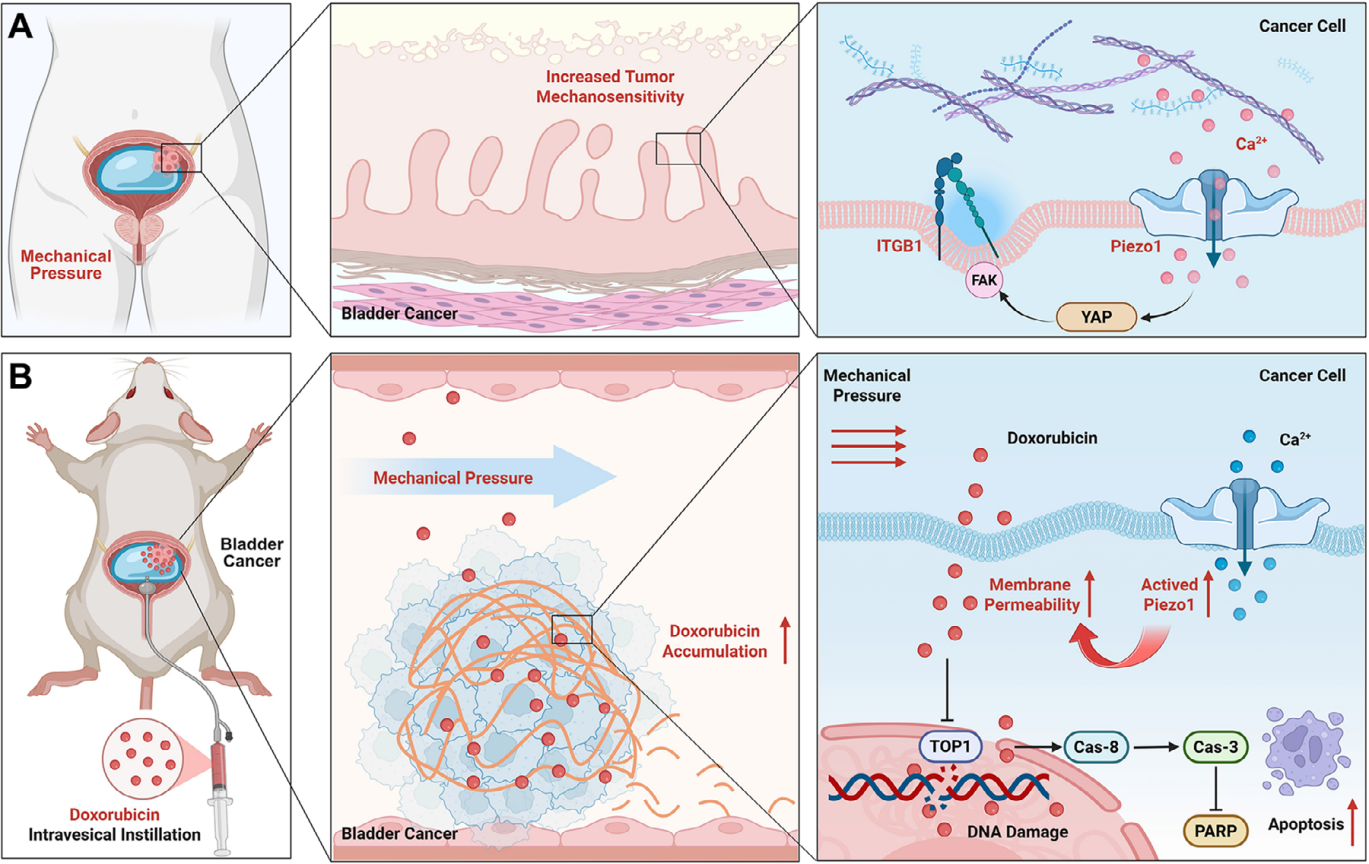
Mechanical pressure unleashes Piezo1 axis to drive mechano‐chemotherapy synergy for intravesical therapy. (A) Mechanical pressure potentiates mechanosensitivity in tumor urothelium via Piezo1/ITGB1 activation. (B) Mechano‐chemotherapy synergistically enhances DOX accumulation and apoptotic execution in tumor area while mitigating dose‐limiting mucosal toxicity.

Here, we decipher a fundamental mechanobiological paradigm in which the Piezo1 channel serves as the master orchestrator of bladder urothelial mechano‐transduction, transforming mechanical forces into biochemical signals that reconfigure cellular architecture and therapeutic responsiveness. Mechanical pressure initially activates the Piezo1, further eliciting rapid calcium influx. This Ca^2^^+^ signal subsequently promotes the activation and clustering of ITGB1, which mediates cellular adhesion and cytoskeletal linkage to sustain Piezo1 activity. Crucially, mechanical force activated the Piezo1 channel to facilitate permeability and accumulation of chemotherapeutics, including DOX and MMC to heighten chemosensitivity in cellular and orthotopic models while mitigating dose‐limiting mucosal toxicity, which establishes a mechano‐chemotherapy strategy to optimize intravesical therapy (Figure 8). By directly and uniformly enhancing drug permeation within the bladder, this strategy can create a more homogeneous drug exposure at the tumor site, overcoming the spatial heterogeneity associated with systemic or passive local delivery of DOX [40]. However, we explicitly note that while our data strongly support a model in which force‐induced penetration is the predominant mechanism possibly, it remains challenging to definitively exclude potential contributions from all forms of endocytosis under these dynamic conditions. Besides, it remains complex to definitively separate cellular uptake of DOX from bulk transport effects within the spectrum of reversible permeability changes because DOX quantification can be confounded by closely related species [41].

Clinically, these findings enhance the effect of intravesical therapy because the bladder's physiological characteristic enables uniform pressure distribution, making it uniquely suited for mechanical intervention [42]. Our orthotopic models further demonstrate that mechanical pressure (40 cm H_2_O) synergizes with 0.5 mm DOX regimens to suppress tumor growth, indistinguishable from 1.0 mm DOX. Meanwhile, it has also been reported that hydrostatic pressure enhances mitomycin C (MMC) induced apoptosis in urothelial carcinoma cells and minimize the side effects deriving from chemotherapy by reducing the concentration of the chemical agent. For clinical cancer treatment, bladder fullness to achieve a specific hydrostatic pressure prior to installation could improve the efficacy of intravesical MMC chemotherapy, but the mechanism still needs to be investigated further [43, 44].

The underlining mechanism of mechanical forces in modulating cellular response and chemotherapy efficacy remains complex. Under elevated hydrostatic pressure, YAP/TAZ regulate rapid, reversible cell volume changes by modulating membrane tension through clathrin‐dependent endocytosis. This mechanoadaptive mechanism enables cells to dynamically respond to fluid pressure fluctuations via Hippo/YAP pathway coordination [38]. Besides, Tan et al. also reveals an inverse correlation between actomyosin‐mediated contractility in tumor cells and their chemosensitivity. It demonstrates that high contractility, propagated through intercellular forces, activates mechanosensitive Notch signaling, which upregulates the downstream effector major vault protein (MVP), facilitating the nuclear export of chemotherapeutic drugs and thereby reducing chemosensitivity. Importantly, inhibiting cellular contractility, Notch signaling, or MVP reversed chemotherapy tolerance and suppressed tumor growth in both cellular and animal models [45]. Therefore, the mechanical signaling pathway is linked to chemotherapy efficacy, suggesting the potential as a novel target for cancer therapeutics [46].

While we establish Piezo1/ITGB1 as the core axis of intravesical instillation, tumor‐specific variations in mechanical axis expression may require more attention. Piezo1 as the key mediator of mechanical‐environmental sensing in cells, not only detects pathogens but also senses hydrostatic pressure, revealing a physiological link between mechanical stimuli and disease progression [47]. The mechanical forces from leukocyte‐induced ICAM‐1 clustering synergize with blood flow‐derived fluid shear stress to elevate endothelial plasma membrane tension, activating the mechanosensitive Piezo1 channel and triggering intracellular calcium influx. This calcium signaling induces phosphorylation of SRC, PYK2, and myosin light chain, ultimately weakening the endothelial barrier to enable leukocyte diapedesis, highlighting the critical cooperation between leukocyte‐generated forces and fluid stress in mechanotransduction‐mediate cell migration [48]. Mechanically activated Piezo1 at glioma cell focal adhesions triggers integrin‐FAK signaling to remodel the extracellular matrix, while increased tissue stiffness reciprocally upregulates Piezo1, creating a pathological feedforward loop [49]. What's more, mechanical pressure originated from urine and TGF‐β1 activate Piezo1 to induce Ca^2+^ influx, calpain2‐mediated talin1 cleavage, and ITGB1 upregulation in tubular cells, driving profibrotic responses and functionally linking mechanical stress to ECM‐integrin remodeling [50]. Our previous study also found that Piezo1/ITGB1 synergizes with Ca^2+^/YAP signaling to propel bladder carcinoma progression via a stiffness‐dependent positive feedback loop [51]. Therefore, mechanochemical therapy achieves cancer therapy while minimizing mucosal toxicity, likely attributable to the lower expression of Piezo1/ITGB1 signaling axis in normal tissues compared to tumor lesions, which reduces their mechanical responsiveness. This differential mechanosensitivity highlights the therapeutic potential of selectively targeting the Piezo1/ITGB1 pathway in malignant tissues, positioning it as a promising tumor‐specific biomarker for precision oncology applications.

Herein, we propose a novel approach that transient bladder distension prior to intravesical instillation primes tumor cells for enhanced drug uptake, effectively improving mechanical delivery and eliminating the dose‐dependent toxicity. Our selected pressure (40 cm H_2_O) is positioned within a physiologically relevant spectrum, because adult bladder pressures during normal filling remain below 40 cm H_2_O and can transiently exceed 100 cm H_2_O during voiding or in pathological states such as BOO. Critically, the controlled pressure is clinically achievable. Existing devices for hyperthermic intravesical chemotherapy (HIVEC) already incorporate temperature and pressure monitoring, providing a direct technology for safe, monitored intravesical pressure modulation. What’ more, Tom Soh et al. have developed a series of nanostructured electrodes enable highly sensitive, real‐time mapping of mechanical forces at the bio‐interface, and offered robust methodology for dynamic, long‐term monitoring of physiological pressure [52, 53]. Future implementations of mechano‐chemotherapy could be significantly optimized by integrating such real‐time, quantitative monitoring technologies to create a feedback‐controlled system for mechano‐chemotherapy strategy.

However, we have to acknowledge that the evaluation of long‐term recurrence, survival, and potential chronic adverse effects like fibrosis was beyond the scope of this murine model, which will be a primary focus in preclinical studies. And it's critical for fully defining the chronic safety and recurrence efficacy profile of this strategy in rats or patient‐derived 3D organoids before clinical translation in the future.

## 4 Conclusion

Our study reports an interesting discovery regarding the role of mechanical force in intravesical chemotherapy of bladder cancer, thereby establishing a mechano‐chemotherapy strategy to optimize intravesical therapy while minimizing the adverse effects to patients. The Piezo1 channel serves as a key mechanosensitive target, enabling urothelial cells to sense mechanical stimuli and drive spatial reorganization of mechanosensitive proteins toward the mucosal epithelium that enhances drug permeability. Mechanical pressure promoted urothelial adhesion, migration, and cellular rigidity, while eliciting transcriptional reprogramming that coordinated cytoskeletal dynamics and calcium signaling, ultimately amplifying membrane tension and potentiating phagocytic function in tumors. Importantly, mechanical activation significantly increased intracellular DOX and MMC delivery, promoting apoptosis in tumor while protecting normal mucosa from chemotherapy‐induced damage. These findings support the clinical translation of mechano‐chemotherapy as a practical strategy to enhance intravesical treatment efficacy and reduce side effects.

## 5 Experimental Section

### 5.1 Materials

Oligonucleotides and primers (Sequences in Table S1) were custom‐synthesized by Sangon Biotech (Shanghai, China). The PrimerScript RT reagent kit and SYBR Green Master Mix were acquired from TAKARA Biotechnology (Dalian, China). EdU Cell Proliferation Kit, Calcein‐AM and Fluo‐4 AM probe were acquired from Beyotime Biotechnology (China). PE Annexin V Apoptosis Detection Kit was acquired from BD Biosciences (USA). Doxorubicin (DOX), Mitomycin C (MMC), Verteporfin, chlorpromazine (CPZ), methyl‐β‐cyclodextrin (MβCD), Dynasore, and CCK‐8 reagent were procured from TargetMol (USA). PIEZO1 agonist (Yoda1), inhibitor (GsMTx4), and EIPA were acquired from MedChemExpress (China). Integrin β1 and PIEZO1 overexpression plasmids were synthesized by Biokeeper (China). Antibodies against Piezo1, ITGB1, YAP1, TOP1, and GAPDH were sourced from Abclonal (China), and α‐SMA, PARP, Caspase‐8, Caspase‐3, Bcl‐2, and Bax antibodies were sourced from Abcam (UK). The Flipper‐TR membrane tension sensor was obtained from Cytoskeleton Inc. (USA). Cell culture media were supplied by Procell Life Science & Technology Co., Ltd. (China). All chemicals were of reagent‐grade purity and used without further purification.

### 5.2 Mechanical Pressure Model in Mice

All experimental protocols were approved by the Animal Ethics Committee of Xi'an Jiaotong University Health Science Center (Approval No. XJTUAE2023‐545) in accordance with institutional and national guidelines for the care and use of laboratory animals. Female BALB/c nude mice (5‐week‐old) were procured from Beijing HFK Bioscience Co. and maintained under specific pathogen‐free conditions. A calibrated piezometer tube was connected to the urinary catheter used for instillation. By infusing saline at incremental volumes into the mouse bladder in anesthetized mice, we recorded the corresponding intravesical pressure in real‐time. Mice were randomized into two cohorts: sham‐treated controls and mechanical pressure groups. The pressure cohort underwent transurethral catheterization to instill 100 µL sterile saline (40 cm H_2_O) into the bladder, maintaining distension for 1 h to simulate intravesical hydrostatic pressure every day. Following this regimen, bladders were harvested after 1 week for further analysis.

### 5.3 Multiplex Immunohistochemistry (mIHC)

Human bladder tissues were collected from normal controls, interstitial cystitis patients, and BOO patients. All clinical tissues and animal tissues were conducted in accordance with the Declaration of Helsinki and approved by the ethics committee of first affiliated hospital of Xi'an Jiaotong University, Xi'an, China (Approval No. LLSBPJ‐2023‐095). Then, paraffin‐embedded sections underwent deparaffinization in xylene substitute and graded ethanol hydration. Antigen retrieval was performed in citrate buffer (pH 6.0) using microwave‐induced epitope retrieval (10 min boiling). Endogenous peroxidase activity was blocked with 3% H_2_O_2_ (25 min, RT) after hydrophobic barrier pen application. Sections were blocked with species‐matched serum (10% rabbit serum for goat primary antibodies, 3% BSA otherwise) for 30 min before sequential incubation cycles: Primary antibody (Piezo1, ITGB1, YAP1, α‐SMA; 4°C overnight) and HRP‐conjugated secondary antibody (50 min, RT) Tyramide signal amplification (iF488‐TSA, iF555‐TSA, or iF647‐TSA; 10 min, RT dark), followed by microwave‐mediated antibody stripping (10 min boiling in citrate buffer). This iterative cycle was repeated for four distinct targets, with the fourth primary detected using iF440‐conjugated secondary antibody (50 min, RT dark). Nuclei were counterstained with DAPI (10 min), autofluorescence quenched (5 min), and sections mounted with anti‐fade medium. Whole‐slide imaging was performed using the digital scanner.

### 5.4 Mechanical Pressure Model In Vitro

The cell culture pressure model was established using MIC‐101 sealed chambers integrated with dual‐flow meters to pre‐equilibrate the gaseous environment. Pressure was applied using a custom‐built hydrostatic pressure system, consisting of a pressure pump connected to the cell culture chamber and a calibrated digital pressure transducer. All pressure applications were performed with cells immersed in complete culture medium and maintained at 37°C. Real‐time pressure monitoring and regulation (0‐200 cm H_2_O) were achieved through a digital manometer‐based feedback system, enabling precise hydrostatic stimulation of cultured cells for different times.

### 5.5 Cell Culture

The human bladder cancer cell lines T24, 253J, and the normal control cell SV‐HUC, which was the SV‐40 immortalized human uroepithelial cell line, were obtained from the American Type Culture Collection (ATCC, USA). SV‐HUC, T24 and 253J cells were cultured in DMEM medium supplemented with 10% FBS under a humidified 5% CO2 and 95% air atmosphere at 37°C.

### 5.6 EdU Assay

Cells were seeded in 24‐well plates with coverslips and cultured overnight to achieve confluency. Following pretreatment with mechanical pressure, cells were pulse‐labeled by adding pre‐warmed 2× EdU working solution to achieve a final 10 µm EdU concentration. After 2‐h incubation at 37°C, cells were fixed with 1 mL paraformaldehyde for 15 min at room temperature. Fixed samples were washed thrice and permeabilized for 15 min (0.3% Triton X‐100 in PBS), and washed twice. The Click reaction mixture was prepared per the manufacturer's specifications. Samples were incubated with 0.5 mL Click reaction solution for 30 min at room temperature, protected from light, followed by three washes. Nuclei were counterstained with 1× Hoechst for 10 min in the dark, washed thrice, and imaged using fluorescence microscopy.

### 5.7 Transmission Electron Microscope (TEM)

T24 cells were treated with hydrostatic pressure for 4 h, washed twice with sterile PBS, and pelleted by centrifugation. Cell pellets were primary‐fixed in 4% (v/v) paraformaldehyde and 1.25% (v/v) glutaraldehyde in 0.1 m cacodylate buffer (pH 7.4) overnight at 4°C, followed by secondary fixation in 2% (w/v) osmium tetroxide for 45 min at room temperature. Samples were dehydrated through an ethanol gradient (50% to 100%), infiltrated with propylene oxide, and embedded in epoxy resin. Polymerized blocks were sectioned at 70 nm thickness using a Leica ultramicrotome, with sections post‐stained with 2% uranyl acetate and Reynolds’ lead citrate. Ultrastructural analysis was performed using a JEM‐1400Plus TEM operating at 100 kV.

### 5.8 Detection of Young's Modulus

Cellular Young's modulus was quantitatively assessed using a Piuma Nanoindenter (Optics11 Life, Netherlands) equipped with a calibrated spherical‐tipped probe (nominal radius: 5 µm, spring constant: 0.08 N/m) mounted on a soft cantilever. Prior to measurement, cells were treated with mechanical pressure for 2 h, and maintained in a minimal volume of pre‐warmed culture medium or CO_2_‐independent medium during indentation within the instrument's fluid cell. The probe was carefully positioned perpendicularly above the nucleus‐adjacent perinuclear region of individual adherent cells visualized via integrated optical microscopy. Force‐indentation curves were acquired under depth‐controlled mode (maximum indentation depth: 2.0 µm, approach/retract velocity: 2.0 µm/s) to ensure minimal perturbation and remain within the linear elastic regime, with a minimum 10 s dwell period at maximum load to mitigate viscoelastic effects. For each experimental condition, at least 16 areas were analyzed. Young's modulus (EPa) was calculated directly from the retract portion of the force curve by fitting the Hertzian contact model for a spherical indenter to the data using the native Piuma Dataview software. Results were presented as mean ± standard deviation, and statistical significance was determined using Student's t‐test based on experimental design.

### 5.9 Western Blotting

Total cellular proteins were extracted using RIPA lysis buffer (Beyotime, China) supplemented with protease inhibitor cocktail, phosphatase inhibitors, and 0.1 m phenylmethylsulfonyl fluoride (PMSF). Following extraction, lysates were centrifuged at 12,000 × g for 15 min at 4°C, and supernatants containing soluble proteins were collected. Protein concentration was determined by bicinchoninic acid (BCA) assay. Equal amounts of protein (20 µg) were resolved by electrophoresis on SDS‐polyacrylamide gels and subsequently transferred to polyvinylidene fluoride (PVDF) membranes. Membranes were blocked for 2 h at room temperature with 5% non‐fat dry milk in Tris‐buffered saline containing 0.1% Tween‐20 (TBST) to prevent nonspecific binding. After blocking, membranes were incubated overnight at 4°C with specific primary antibodies, followed by three 10‐min washes with TBST. Immunodetection was performed by incubating membranes with horseradish peroxidase (HRP)‐conjugated secondary antibodies for 1 h at room temperature. Following additional TBST washes, protein expression was visualized using an Enhanced Chemiluminescence (ECL) detection system (Bio‐Rad, USA).

### 5.10 Calcium Ion Analysis

SV‐HUC, T24 and 253J cells were treated with mechanical pressure for different times, washed thrice with phosphate‐buffered saline (PBS), and subsequently loaded with 2 µm Fluo‐4 AM calcium indicator. Cells were incubated with the dye at 37°C for 30 min in dye‐containing medium to facilitate cellular uptake and intracellular hydrolysis to its calcium‐sensitive form. Following three additional PBS washes to remove extracellular dye, cells were equilibrated in fresh medium for 20 min at 37°C. Intracellular calcium dynamics were then quantified via flow cytometry and visualized by fluorescence microscopy.

### 5.11 Fluorescence Lifetime Imaging Microscopy (FLIM)

Cells seeded on glass‐bottom dishes were treated with mechanical pressure for 2 h prior to labeling. Flipper‐TR probe was dissolved in DMSO and diluted to 500 nm in serum‐free medium. Cells were incubated with the probe for 30 min at 37°C under 5% CO_2_, followed by three washes with pre‐warmed PBS to remove excess dye. FLIM was performed on a Leica confocal system equipped with a 488‐nm pulsed white‐light laser (80 MHz repetition rate). Time‐correlated single‐photon counting (TCSPC) data were acquired with photon counts >10^6^ per pixel. Lifetime decay curves were analyzed via SymPhoTime 64 software using bi‐exponential fitting: τ_1_ (short component, instrument response) and τ_2_ (membrane tension‐sensitive component). The amplitude‐weighted mean lifetime (τ_m_ = α_1_τ_1_ + α_2_τ_2_) was calculated, with decreased τ_2_ values indicating increased membrane tension.

### 5.12 Uptake Efficiency of DOX

DOX uptake efficiency was quantified by flow cytometry. Cells seeded in 6‐well plates (1.0 × 10^5^ cells/well) were treated for 4 h at 37°C in medium with DOX, mechanical pressure, and specific endocytosis inhibitors: methyl‐β‐cyclodextrin (MβCD, 5 µg/mL; lipid raft inhibition), EIPA (10 µm; macropinocytosis inhibition), chlorpromazine (CPZ, 5 µg/mL; clathrin‐mediated endocytosis inhibition), or dynasore (80 µm; dynamin‐dependent endocytosis inhibition). Following treatment, cells were trypsinized, washed twice with ice‐cold PBS, and resuspended in 1 mL PBS. Fluorescence intensity of cellular internalization was measured for 10,000 events per sample using a BD FACS flow cytometer.

### 5.13 3D Tumor Spheres

3D tumor spheroids were generated by suspending T24 cells in methylcellulose‐supplemented DMEM (0.12% w/v) at 1 × 10^6^ cells/mL. Twenty‐five microliter aliquots of cell suspension were dispensed onto the inverted lid of a 100‐mm culture dish to form uniform hanging droplets. The base chamber was filled with 10 mL sterile PBS to maintain humidity. Following 72‐h incubation (37°C, 5% CO_2_), compact spheroids were individually transferred to ultra‐low attachment 96‐well plates and cultured for an additional 72 h. Mature spheroids were then subjected to either 0.5, 1.0 µm DOX exposure, 40 cm H_2_O hydrostatic pressure, or combinatorial treatment for 4 h. Processed spheroids were harvested for subsequent confocal immunofluorescence analysis. The expression of Piezo1/ITGB1 mechanoreceptors and DOX accumulation was quantified using ImageJ software.

### 5.14 Immunofluorescence

SV‐HUC, T24, 253J cells or 3D tumor spheres were seeded onto glass coverslips and treated with mechanical pressure and DOX. Following fixation and permeabilization, cells were incubated overnight at 4°C in a humidified chamber with primary antibodies against ITGB1 and Piezo1 (1:200 dilution). After thorough washing, coverslips were incubated with appropriate fluorophore‐conjugated secondary antibodies for 1 h at room temperature. Nuclei were counterstained with DAPI for 5 min at room temperature. Coverslips were mounted using anti‐fade mounting medium and imaged by fluorescence microscopy to visualize target protein localization.

### 5.15 Apoptosis Analysis

Apoptosis rates in SV‐HUC, T24 and 253J cells treated with mechanical pressure and DOX were quantified by flow cytometry. Cells were washed twice with ice‐cold phosphate‐buffered saline (PBS), centrifuged at 500 g for 5 min at 4°C, and resuspended in 100 µL binding buffer. Subsequently, cells were stained with 4 µL Annexin V‐fluorescein isothiocyanate (FITC) and propidium iodide (PI), followed by 15‐min incubation in darkness at room temperature. Samples were then diluted with 400 µL binding buffer and immediately analyzed by flow cytometry to determine apoptotic populations.

### 5.16 Uptake Assay for Membrane Permeability

To assess non‐selective membrane permeability, a calcein acetoxymethyl (Calcein‐AM) uptake assay was performed. Briefly, T24 cells were seeded in plates overnight. Cells were divided into different treatment groups for 1 h: Control, Piezo1 agonist Yoda1 (5 µm), Piezo1 inhibitor GsMTx4 (2.5 µm), HP (40 cm H_2_O), HP‐Yoda1, HP‐GsMTx4. Immediately following the respective treatments, cells were incubated with 1 µm Calcein‐AM in serum‐free medium for 20 min at 37°C. The cells were then washed three times with ice‐cold PBS to remove extracellular dye, and fluorescence was visualized under a fluorescence microscope. Each experiment was performed with at least three independent replicates.

### 5.17 Reversibility of Pressure‐Induced Permeability

To evaluate the reversibility of the mechanical effect, cells were subjected to hydrostatic pressure (40 cm H_2_O) for 1 h. Following pressure release, cells were returned to standard culture conditions. Membrane permeability was assessed at different recovery time points (0, 0.5, 1, 2, and 4 h) using the Calcein‐AM uptake assay as described above. The 0‐h time point was measured immediately after pressure cessation without a recovery period.

### 5.18 Orthotopic Bladder Tumor‐Bearing Mice

All animal procedures were approved by the Xi'an Jiaotong University Animal Ethics Committee (Approval No. XJTUAE2023‐545) and conducted in accordance with institutional guidelines. Female nude mice (4–6 weeks old) were randomized, anesthetized via isoflurane inhalation, and catheterized transurethrally using a paraffin‐lubricated 24‐gauge intravenous catheter. After complete bladder voiding and triple PBS lavage, a 1‐cm midline laparotomy was performed under aseptic conditions. T24 cells (1 × 10^3^ cells in 100 µL PBS) were orthotopically implanted into the bladder wall using insulin syringe under direct visualization. The abdominal wall was closed with silk sutures, and a sterile dressing was applied. Tumor establishment was confirmed 7 days post‐implantation. Treatment cohorts received intravesical perfusion for 1 h of: (i) 0.5 mm DOX, (ii) 0.5 mm DOX after hydrostatic pressure (40 cm H_2_O) for 1 h, or (iii) 1.0 mm DOX. In vivo biodistribution was quantified at 5, 10, 15 days using an AniView IVIS imaging system. Tumors were subsequently excised, cryosectioned, and counterstained with DAPI for fluorescence microscopic analysis. Systematic quantification of drug distribution in tumor area was performed using ImageJ software.

### 5.19 Immunohistochemical Staining

Paraffin‐embedded tissue sections underwent sequential dewaxing in xylene (3 × 10 min), rehydration through a graded ethanol series, and distilled water rinsing. Antigen retrieval was performed in preheated citrate buffer (10 mm, pH 6.0) using pressurized heating at 95°C for 20 min followed by ambient cooling. Endogenous peroxidase activity was quenched with 3% H_2_O_2_/methanol (15 min, RT). Non‐specific binding was blocked with 5% BSA (1 h, 37°C). Sections were incubated overnight at 4°C with primary antibodies against Caspase ‐ 3 and Bcl ‐ 2 (1:200). After PBS washing, HRP‐conjugated secondary antibodies were applied (1 h, RT). Signal development utilized a DAB substrate kit with real‐time microscopic monitoring to optimize chromogen exposure, immediately terminated by distilled water rinsing. Counterstaining employed Mayer's hematoxylin (1 min) with differentiation in 1% acid alcohol and bluing in 0.2% ammonia water. Sections were dehydrated through an ascending ethanol series, cleared in xylene, and mounted with neutral balsam. Whole‐slide imaging was performed at consistent optical settings, followed by batch‐processed quantitative analysis of staining intensity and positive‐cell percentage across multiple representative fields using ImageJ.

### 5.20 TUNEL Staining

Paraffin‐embedded tissue sections were dewaxed in xylene and rehydrated through graded ethanols, followed by distilled water rinsing. Antigen retrieval was performed using preheated citrate buffer (10 mm, pH 6.0) under pressurized heating at 95°C for 20 min. After cooling and PBS washing, sections were permeabilized with 20 µg/mL Proteinase K (15 min, 37°C) and endogenous peroxidase blocked with 3% H_2_O_2_ (10 min, RT). Following PBS washes, sections were equilibrated in TdT buffer (30 min, RT) and incubated with TUNEL reaction mixture containing terminal deoxynucleotidyl transferase (TdT) and fluorescein‐dUTP (60 min, 37°C in humidified darkness). For enzymatic detection, anti‐fluorescein‐HRP conjugate (30 min, 37°C) was applied after TUNEL reagent removal. DAB chromogen development was microscopically monitored, immediately halted by distilled water rinsing. Sections were counterstained with Mayer's hematoxylin, dehydrated through graded ethanols, cleared in xylene, and mounted with resinous medium. Whole‐slide digital imaging was employed and analyzed by ImageJ software.

### 5.21 Statistical Analysis

Data were expressed as the means ± standard deviation (SD). Significant differences were determined using Student's t‐test or one‐way ANOVA as appropriate. A 2‐tailed p < 0.05 was considered statistically significant. ^*^ *p* < 0.05; ^**^ *p* < 0.01; ^***^ *p* < 0.001; ^****^ *p* < 0.0001 (*n* = 3). All data were analyzed with GraphPad Prism, ImageJ and SPSS software.

## Author Contributions

M.M. performed conceptualization, data curation, methodology, validation, and wrote the original draft. X.L. and M.J. performed data curation, methodology, software, and visualization. Z.Y., J.H., J.L., X.L., and Y.J. performed data curation, methodology and investigation. R.H., Y.Z., Y.P., J.X., Y.C., K.M., and P.Z. performed software and visualization. L.W. and J.F. performed supervision, project administration, funding acquisition, review and edited the final manuscript.

## Conflicts of Interest

The authors declare no conflicts of interest.

## Supporting information




**Supporting File**: advs74681‐sup‐0001‐SuppMat.docx.

## Data Availability

The data that support the findings of this study are available from the co‐responding author upon reasonable request.
