# Supplementary material for: Piezo1 Channel Mediates Mechanically Programmable Drug Delivery to Potentiate Intravesical Chemotherapy
Source: Adv Sci (Weinh). 2026 Mar 4;13(27):e22936. doi: 10.1002/advs.202522936 (PMC13170195; doi:10.1002/advs.202522936)
Supplement: Supplementary file 1 — Supporting File: advs74681‐sup‐0001‐SuppMat.docx. [file ADVS-13-e22936-s001.docx]

**Piezo1 Channel Mediates Mechanically Programmable Drug Delivery to Potentiate Intravesical Chemotherapy**

Minghai Ma, Xing Li, Minxuan Jing, Zezhong Yang, Jiale He, Jianpeng Li, Xiao Liang, Yunzhong Jiang, Rou Huang, Yuanquan Zhang, Yuanchun Pu, Jiawei Xiu, Yutong Chen, Kaibo Mi, Pu Zhang, Lei Wang^*^, Jinhai Fan^*^

M. Ma, M. Jing, Z. Yang, J. He, J. Li, Y. Jiang, Y. Pu, Y. Chen, K. Mi, J. Fan

Department of Urology

The First Affiliated Hospital

Xi’an Jiaotong University

Xi’an 710061, China.

Email: fanjinhai@xjtu.edu.cn

M. Ma, X. Li, X. Liang, R. Huang, Y. Zhang, J. Xiu, L. Wang

Department of Thoracic Surgery

Tangdu Hospital,

Air Force Medical University

Xi’an 710038, China.

Email: [tuodi86@fmmu.edu.cn](mailto:tuodi86@fmmu.edu.cn)

P. Zhang

Department of Urology

Zhejiang Provincial People’s Hospital,

Hangzhou Medical College

Hangzhou 314408, China.

**Table S1.** The Gene-specific primers used in the study.

| ITGB1 Reverse | CCTTTGCTACGGTTGGTTACATT |
| --- | --- |
| ITGB1 Forward | CCTACTTCTGCACGATGTGATG |
| Piezo1 Reverse | GGGCACAATATGCAGGCAGA |
| Piezo1 Forward | GGACTCTCGCTGGTCTACCT |
| YAP1 Reverse | TCATGCTTAGTCCACTGTCTGT |
| YAP1 Forward | TAGCCCTGCGTAGCCAGTTA |
| α-SMA Reverse | GCCATGTTCTATCGGGTACTTC |
| α-SMA Forward | AAAAGACAGCTACGTGGGTGA |
| 18S Reverse | TAGTAGCGACGGGCGGTGTG |
| 18S Forward | CAGCCACCCGAGATTGAGCA |


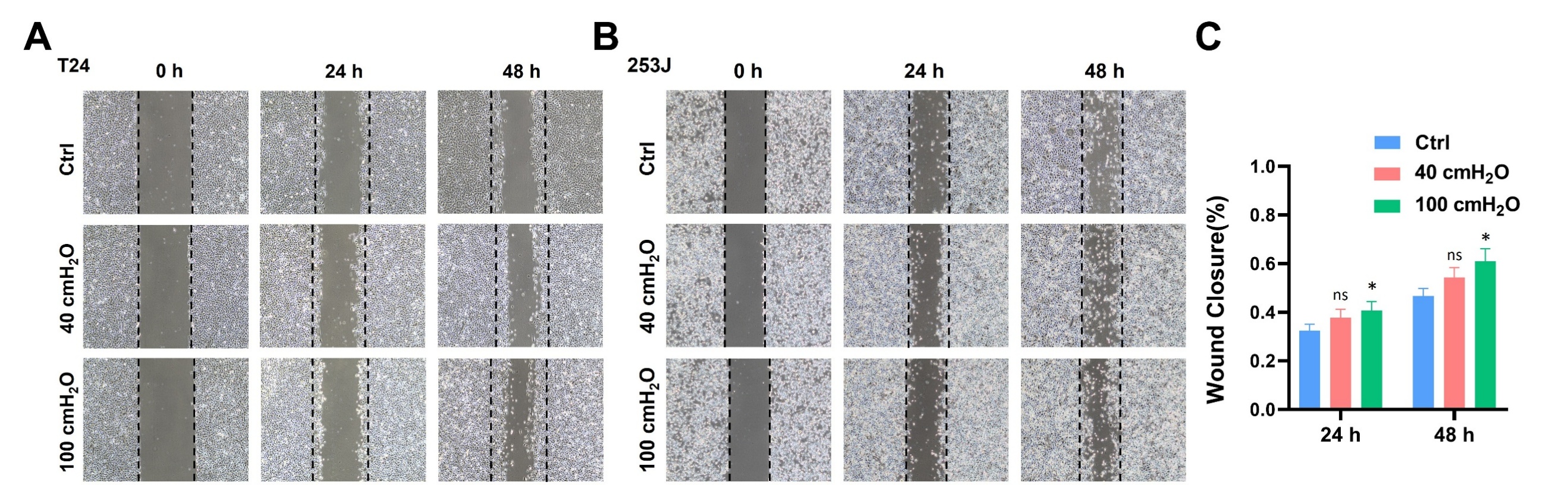


**Figure S1**. A) The impact of mechanical pressure on T24 tumor cell motility. B) The impact of mechanical pressure on 253J tumor cell motility. C) The quantitative data of wound healing result. All data are presented as the Mean ± SD (n = 3). * *p <* 0.05, ** *p <* 0.01, and *** *p <* 0.001. ns, no significant difference.


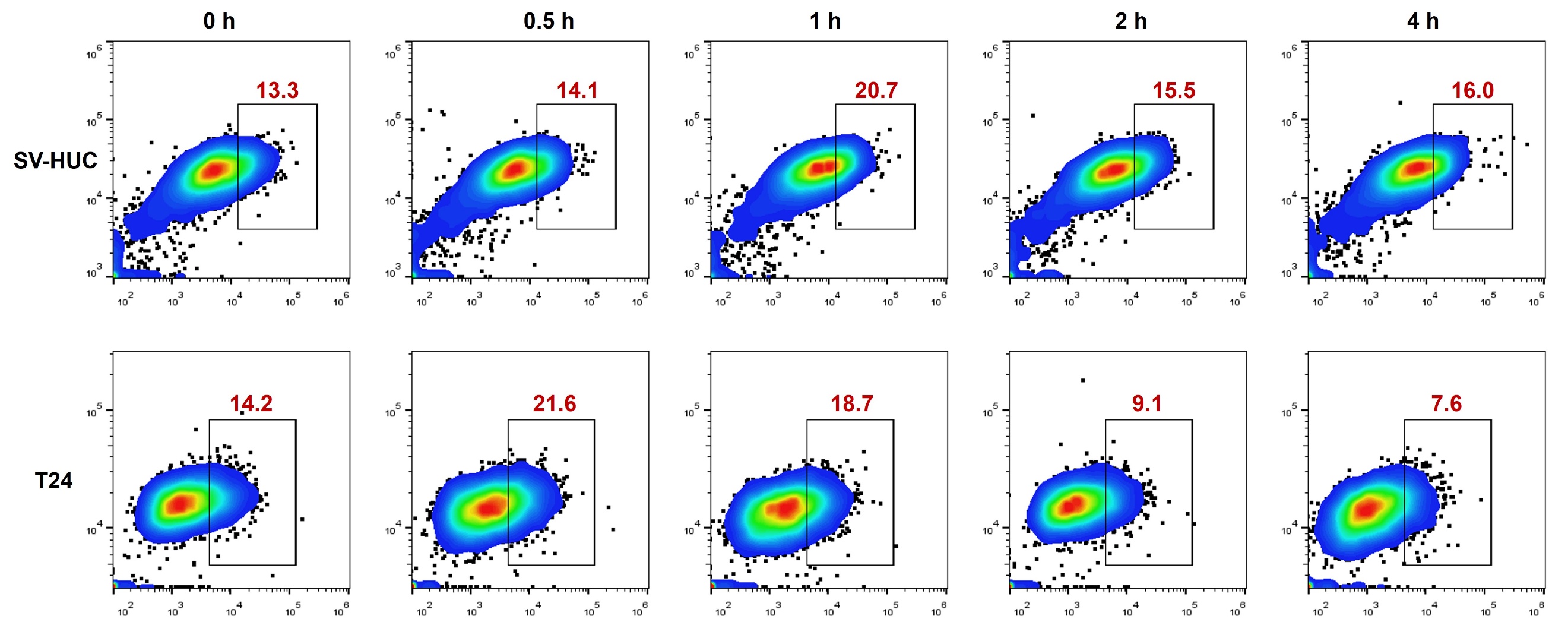


**Figure S2**. Flow cytometry results of cellular Ca^2+^ influx in SV-HUC and T24 cells exposed on HP for 0, 0.5, 1, 2, 4 h.


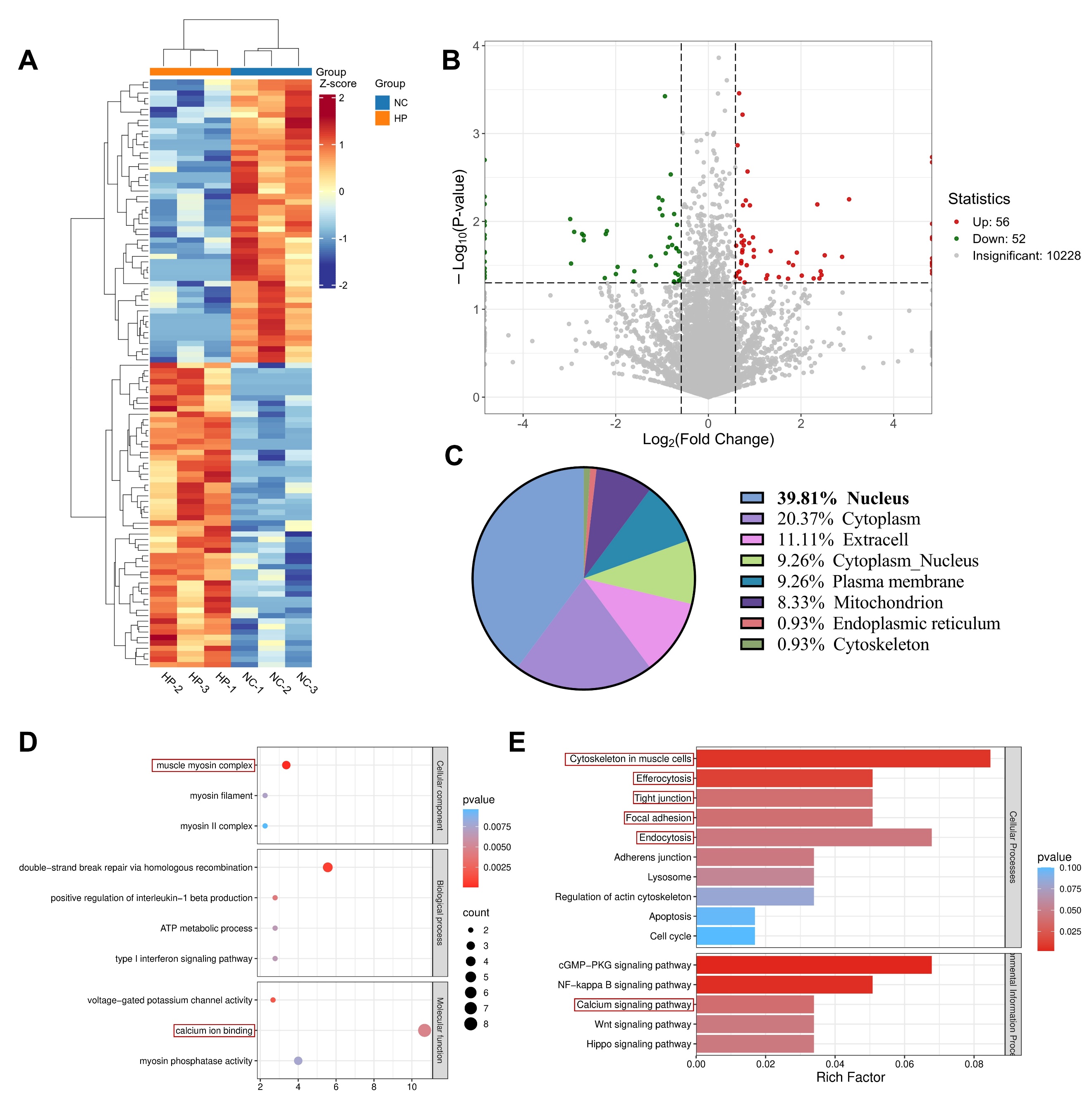


**Figure S3**. A) The heatmap of differentially expressed proteins (DEPs) in pressure-treated cells. B) The volcano plot of differentially expressed proteins (DEPs) in pressure-treated cells. C) The cellular location of DEPs in pressure-treated cells. D) GO analysis of functional annotation in DEPs. (E) KEGG analysis of pathway annotation in DEPs.


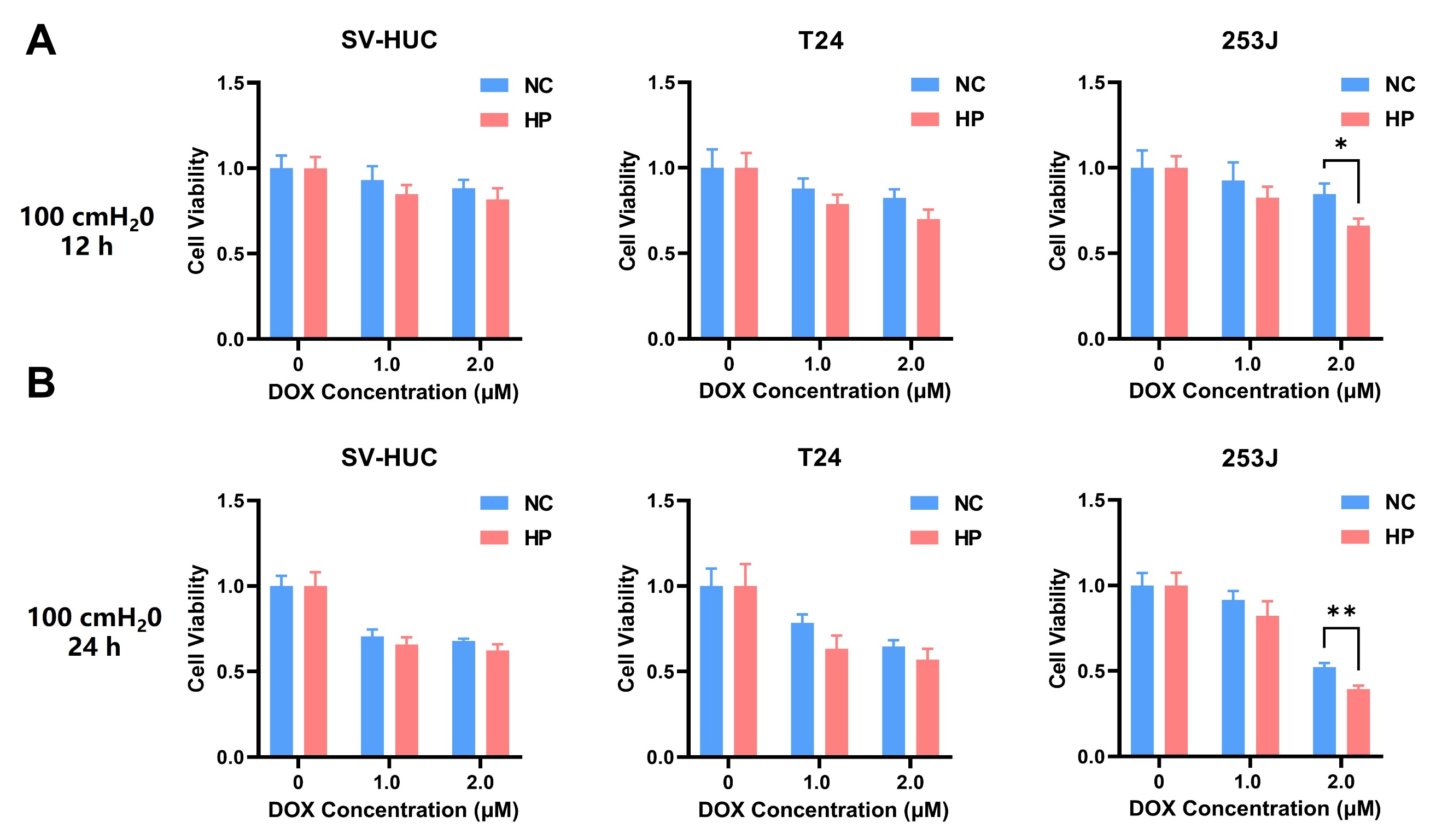


**Figure S4**. A) The viability of T24 cells treated with different concentrations of DOX under 100 cmH_2_O for 12 h. B) The viability of T24 cells treated with different concentrations of DOX under 100 cmH_2_O for 24 h.


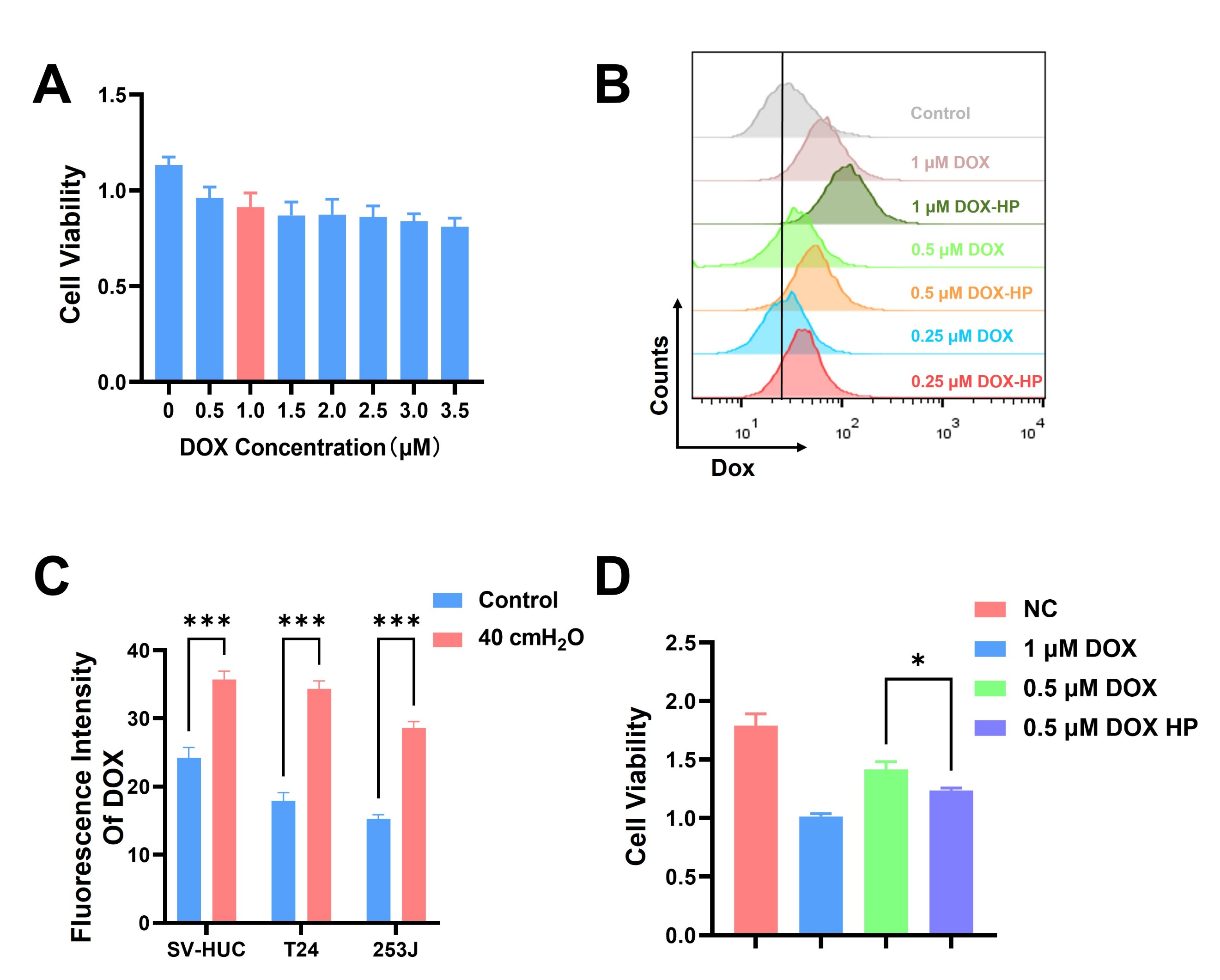


**Figure S5.** A) The viability of T24 cell treated with different concentrations of DOX. B) The uptake efficiency of T24 cell treated with different concentrations of DOX and hydrostatic pressure. C) The quantitative uptake efficiency of cells treated with DOX and hydrostatic pressure from flow cytometry in Figure 4B. D) The quantitative cell viability of cells treated with DOX and hydrostatic pressure. All data are presented as the Mean ± SD (n = 3). * *p <* 0.05, ** *p <* 0.01, and *** *p <* 0.001. ns, no significant difference.


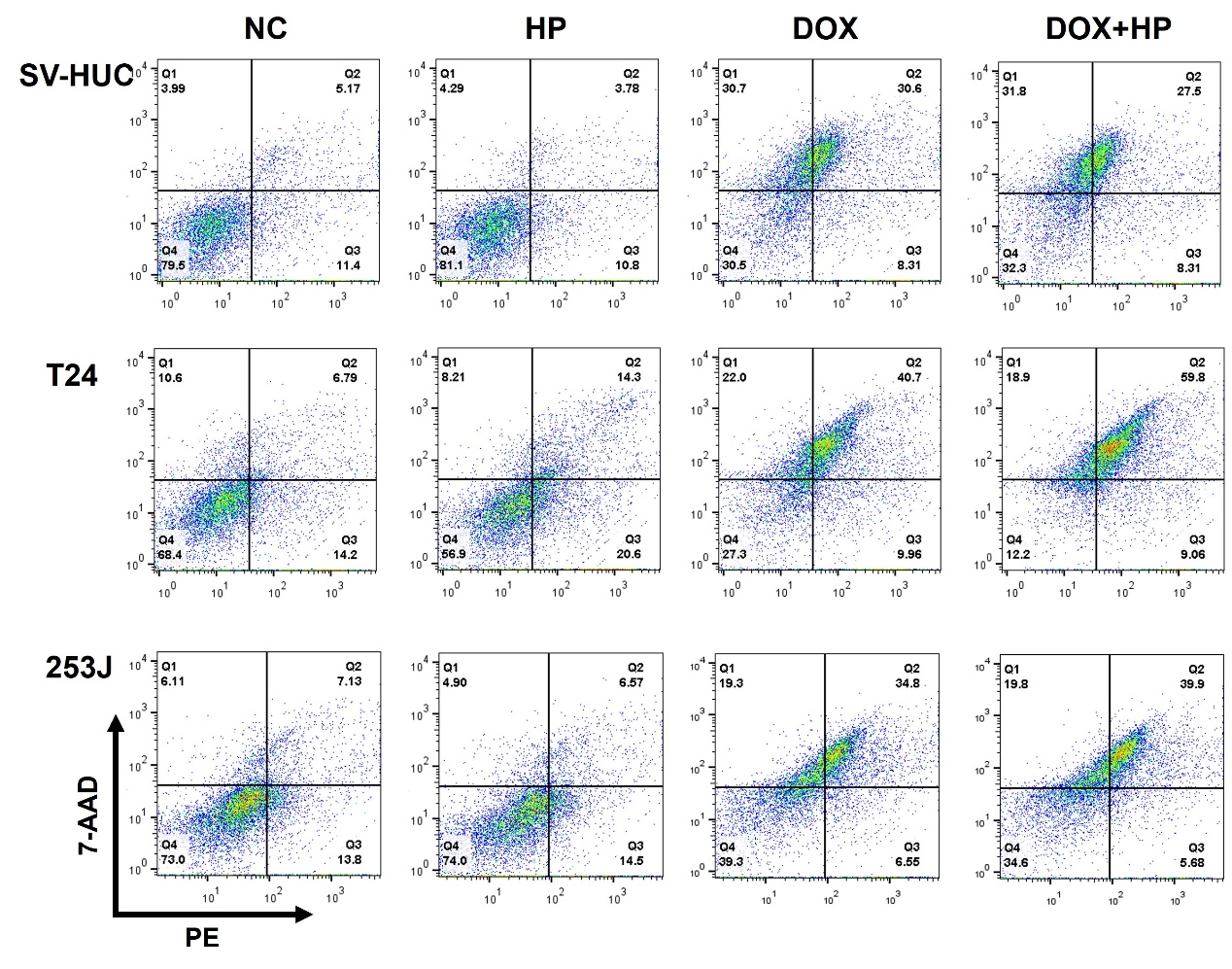


**Figure S6**. The apoptosis rate of SV-HUC, T24, 253J cells induced by DOX and hydrostatic pressure from flow cytometry.


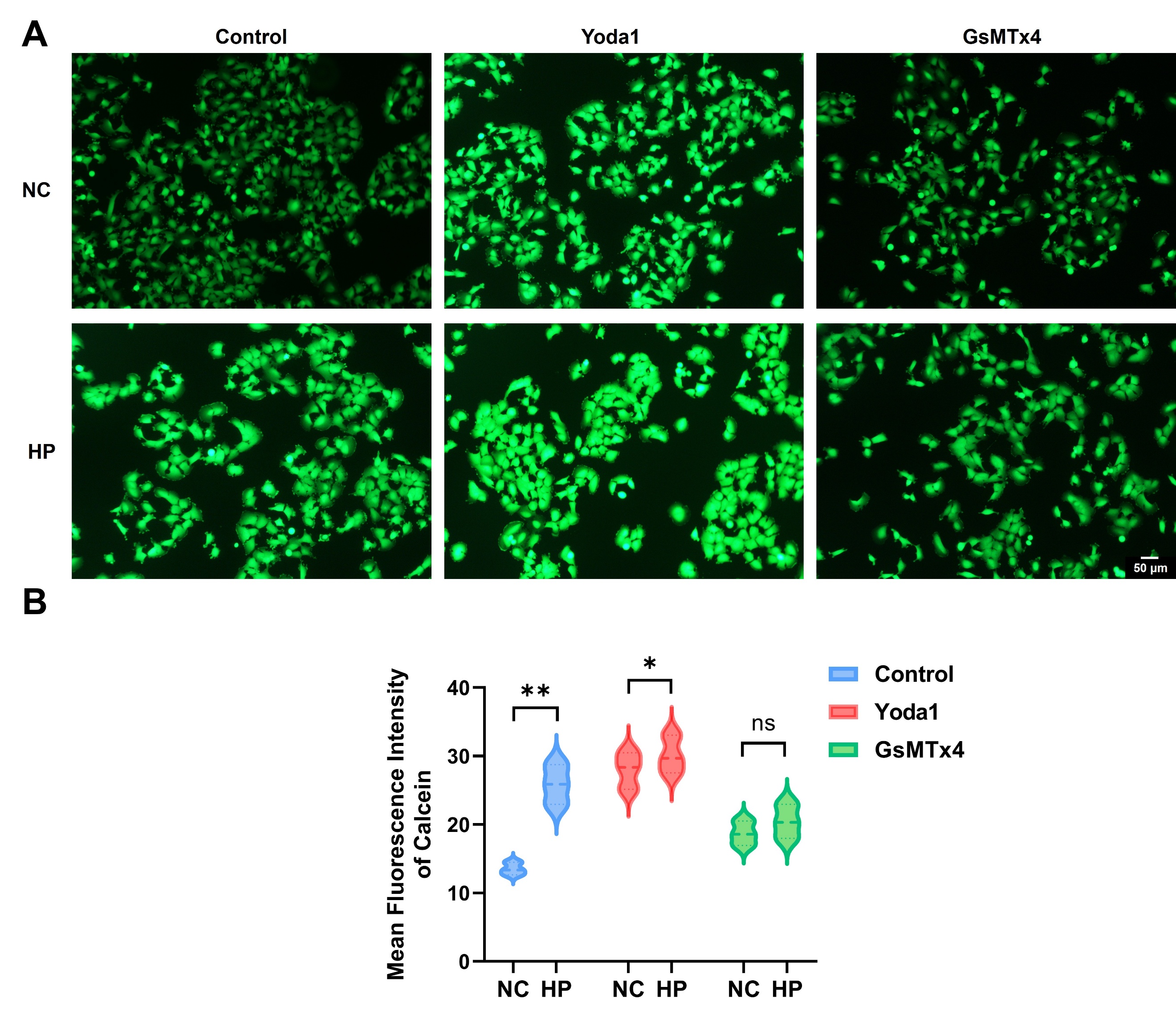


**Figure S7**. A) Calcein-AM uptake efficiency in T24 cells exposed on HP and Piezo1 agonist (Yoda1) and inhibitor (GsMTx4) for 1 h. B) The quantitative result of Calcein-AM uptake efficiency. All data are presented as the Mean ± SD (n = 3). * *p <* 0.05, ** *p <* 0.01, and *** *p <* 0.001. ns, no significant difference.


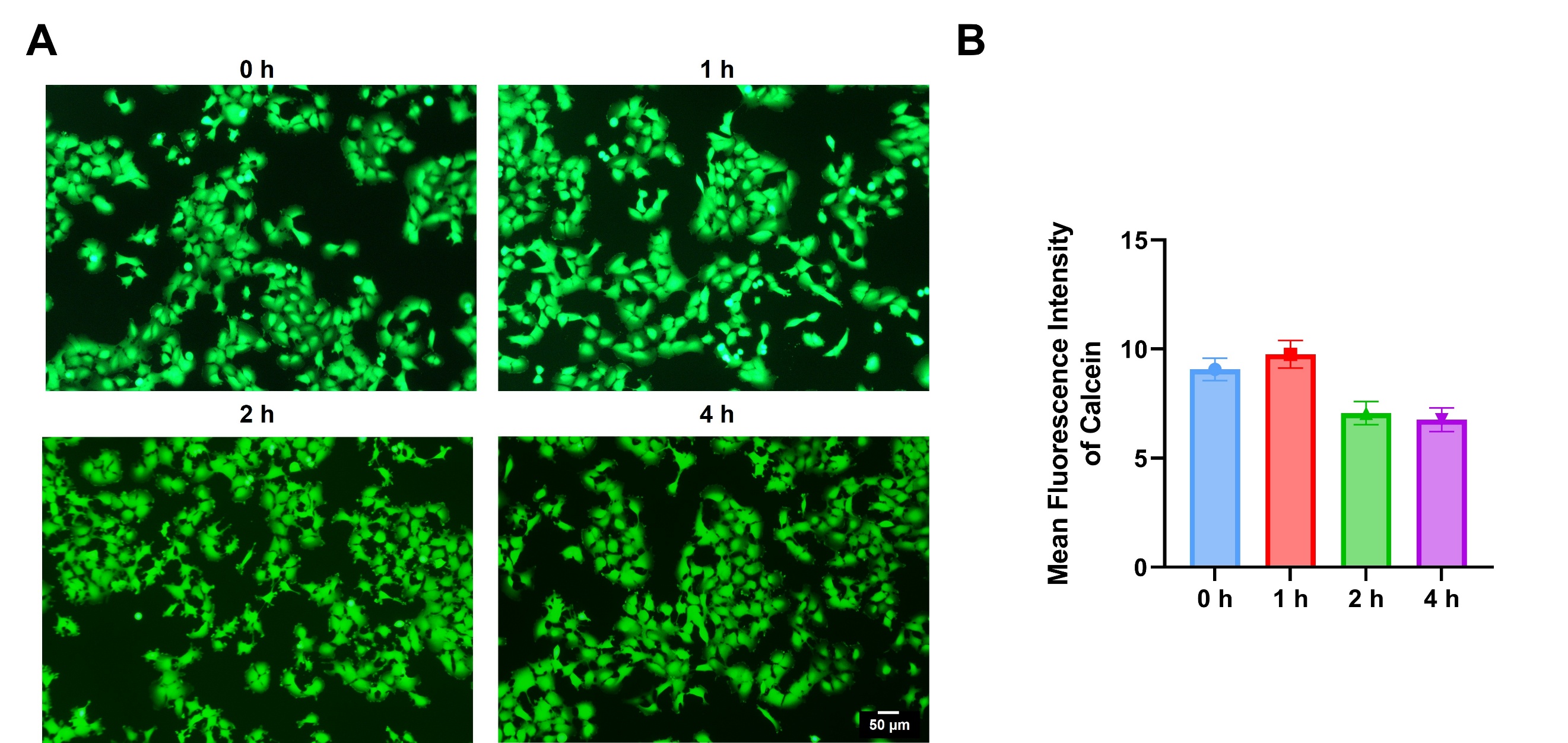


**Figure S8**. A) Calcein-AM uptake efficiency in T24 cells after HP treatment for 0, 1, 2, 4 h. B) The quantitative result of Calcein-AM uptake efficiency.


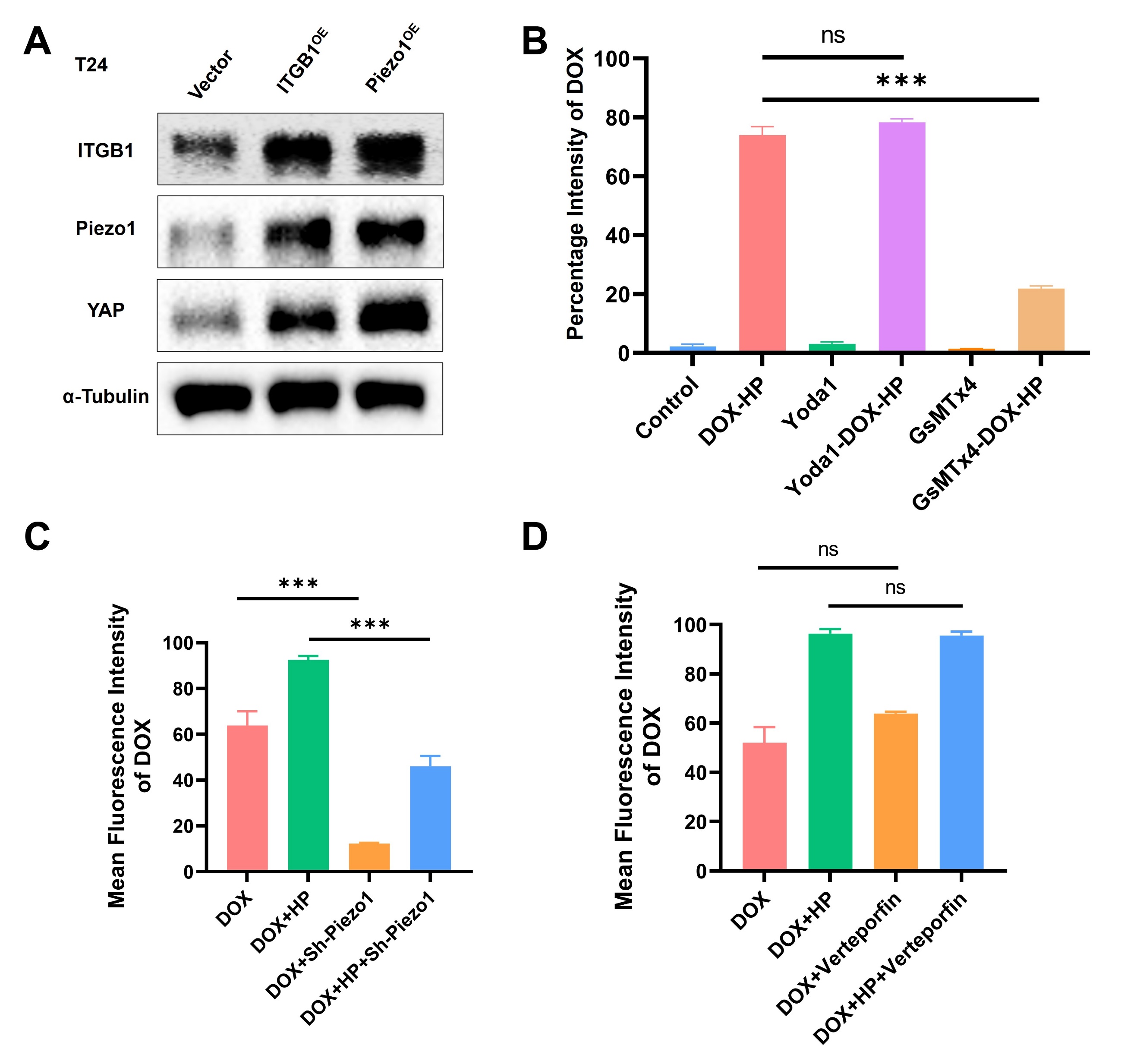


**Figure S9**. A) The expression level of Piezo1/ITGB1/YAP in T24 cell overexpressed Piezo1 or ITGB1 via lentiviral transduction. B) The quantitative result of DOX accumulation in T24 cell exposed on HP with Piezo1 agonists or inhibitors from flow cytometry. C) The quantitative result from flow cytometry of DOX accumulation in Sh-Piezo1 cells exposed on HP. D) The quantitative result from flow cytometry of DOX uptake in T24 cells treated with YAP-specific inhibitor verteporfin. All data are presented as the Mean ± SD (n = 3). * *p <* 0.05, ** *p <* 0.01, and *** *p <* 0.001. ns, no significant difference.


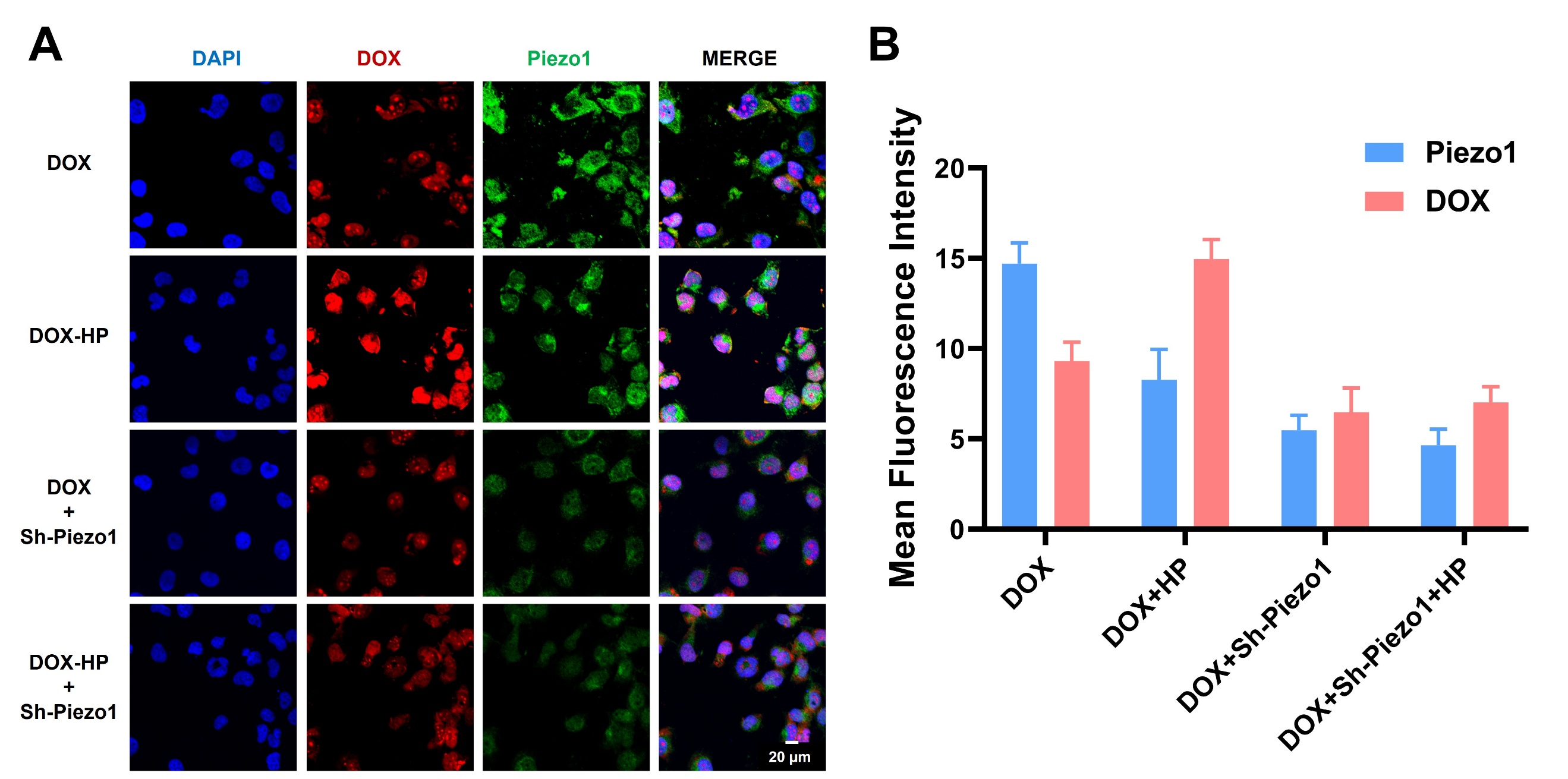


**Figure S10.** A) Immunofluorescence of Piezo1 and DOX in T24 cell knocked down Piezo1. B) The quantitative analysis of Piezo1 expression and DOX nuclear accumulation in T24 cell.


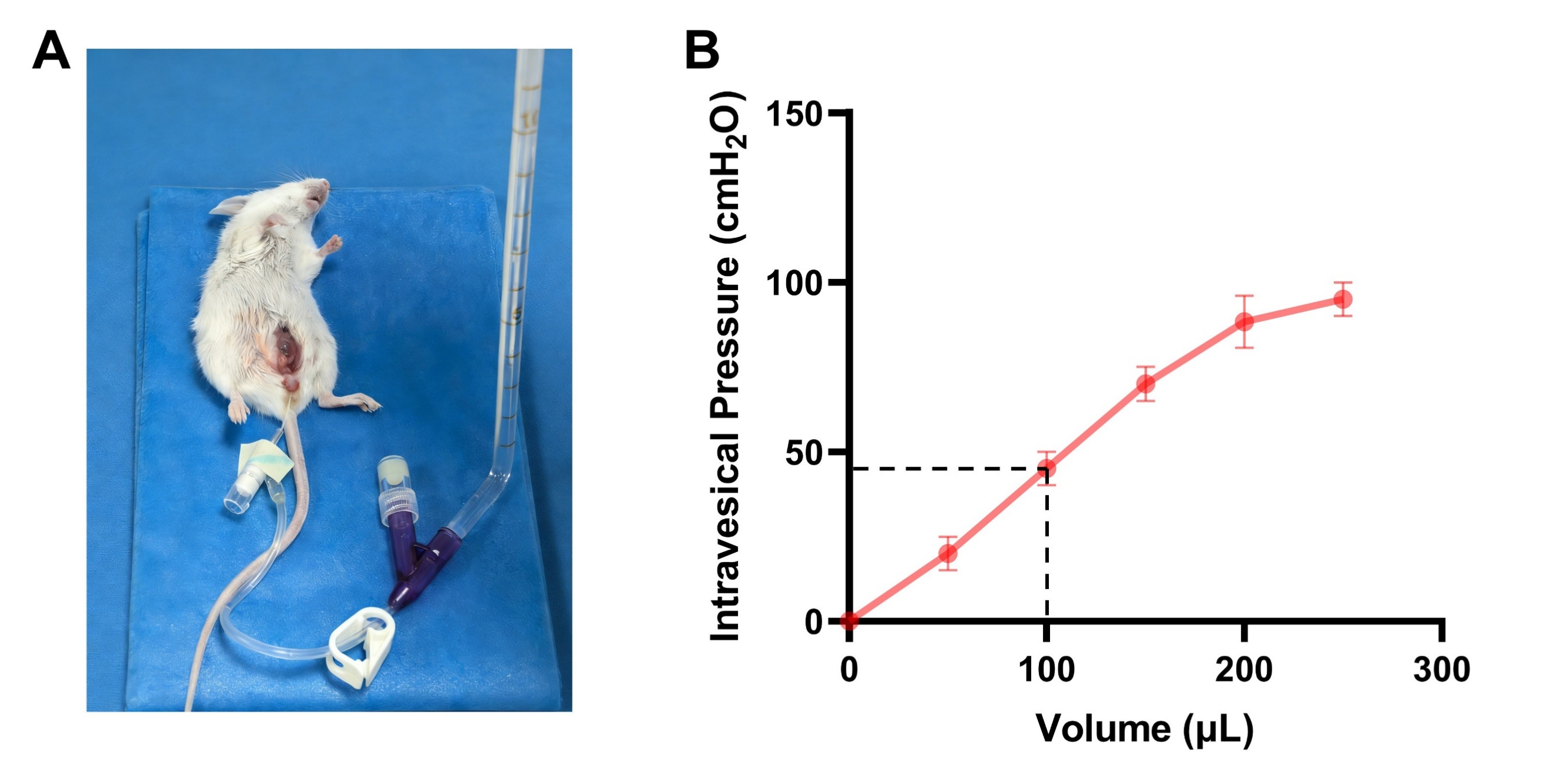


**Figure S11**. A) The image of murine bladder pressure measurement. B) The curve of murine bladder pressure-volume relationship.
